# Exogenous Nucleotides Supplementation Attenuates Age‐Related Sarcopenia

**DOI:** 10.1002/jcsm.70002

**Published:** 2025-07-31

**Authors:** Xin Wu, Rui Liu, Na Zhu, Xiujuan Wang, Chan Wei, Xiaoyang An, Meihong Xu, Yong Li

**Affiliations:** ^1^ Department of Nutrition and Food Hygiene, School of Public Health Peking University Beijing China; ^2^ Beijing Key Laboratory of Protein Posttranslational Modifications and Cell Function, Department of Biochemistry and Molecular Biology, School of Basic Medical Science Peking University Beijing China; ^3^ College of Public Health Inner Mongolia Medical University Hohhot China

**Keywords:** ageing, FoxO, muscle, SAMP8 mice, sarcopenia

## Abstract

**Background:**

Sarcopenia, an age‐related clinical syndrome characterized by reduced skeletal muscle mass and strength, often leads to a loss of physical function. Nucleotides (NTs) supplementation is a potential strategy for preventing age‐related sarcopenia as evidence suggests that NTs declined in muscles with aging, and 5′‐CMP, 5′‐UMP can mitigate muscle atrophy in C2C12 myotubes. This study aimed to investigate the effects of NTs supplementation on sarcopenia and its possible mechanism.

**Methods:**

Senescence‐accelerated mouse prone‐8 (SAMP8) mice and C2C12 cells were used to assess the effect of NTs on sarcopenia. We fed an NTs‐enriched mixture to SAMP8 mice starting from the age of 3 months for 9 months or 15 months. NTs' effects on H_2_O_2_‐induced C2C12 atrophy cells were tested using nucleotide monomers and mixtures. The body composition was measured by EchoMRI analysis meter. Physical performance was tested including grip strength, wire hang, horizontal bar and gait test. The immunofluorescence staining was used to evaluate the cross‐sectional area (CSA) or type of muscle fibers and the diameter of myotubes. RT‐qPCR, western blot, RNA sequencing (RNA‐seq) and targeted metabolomic analyses were conducted to elucidate the underlying mechanisms.

**Results:**

The results demonstrated that NTs significantly increased lean mass/body weight (*p* < 0.01, *η* ^2^ = 0.434), the grip strength at 7 (*p* < 0.0001, *η* ^2^ = 0.312), 9 (*p* < 0.001, *η* ^2^ = 0.293) and 11 (*p* < 0.0001, *η* ^2^ = 0.507) months of age and the gait speed of mice (*p* < 0.0001*, η* ^2^ = 0.3861). The immunofluorescence staining results indicated that NTs increased the CSA of muscle fibers (*p* < 0.0001, *η* ^2^ = 0.1081), especially for type IIb fibres. The RNA‐seq results showed that NTs significantly downregulated the expression of sarcopenia‐related genes (Trim63, Dkk3 and Mt1). The downregulation of Fbxo32, Trim63, Dkk3, Mt1 and p53 genes in NTs intervention group was confirmed by RT‐qPCR and/or western blot (*p* < 0.05). Integrated analyses of RNA‐seq and metabolomic showed that NTs could cause changes in metabolites, such as ketoleucine, 3‐hydroxylisovalerylcarnitine and 3‐methyl‐2‐oxovaleric acid, which could further regulate sarcopenia‐related genes and inhibit protein degradation and promote protein synthesis. In vitro studies confirmed that NTs increased myotubes diameter and decreased expression of sarcopenia‐related genes.

**Conclusions:**

Our findings reveal the protective role of NTs in improving muscle protein balance during ageing, suggesting they may serve as a conditionally essential nutrient for older individuals. Further research is needed to explore the efficacy and safety of NTs supplementation for sarcopenia in humans.

## 1 Introduction

Sarcopenia is an age‐related syndrome marked by reduced muscle mass, strength and physical function, often leading to adverse outcomes like falls, fractures and mortality [1]. With the increase of population ageing, the prevalence of sarcopenia is increasing rapidly, affecting approximately 5%–13% of individuals over 65 years and as high as 50%–60% among those over 80 [2]. The occurrence and development of sarcopenia are affected by many factors such as age, nutritional status, physical activity and hormones. Currently, there are no specific pharmacological treatments for sarcopenia. Nutrition intervention and physical exercise are recommended prevention and treatment strategies for sarcopenia [3] (Reference S1). However, exercise can be challenging for elderly individuals with low physical function. Therefore, exploring effective nutritional interventions to slow down sarcopenia is of significant scientific value.

Skeletal muscle is the primary protein reserve in the human body, storing about 60% of the body's protein (Reference S2). Maintaining skeletal muscle mass relies on a balance between anabolic and catabolic processes. Sarcopenia results from an imbalance between these processes [4]. The ubiquitin–proteasome system (UPS) is a major protein degradation pathway, playing a crucial role in muscle atrophy development [5]. Fbxo32 (Atrogin1/MAFbx) and Trim63 (MuRF1) are specific ubiquitin ligases in skeletal muscle that regulate protein degradation [6] (Reference S3) and are key markers of muscle atrophy (Reference S4). During muscle atrophy, the expression levels of Trim63 and Fbxo32 are significantly increased. FoxO3 and FoxO1, members of the forkhead transcription family, can activate Fbxo32 and Trim63, causing muscle atrophy [7] (Reference S5). Transgenic mice with muscle‐specific overexpression of FoxO3 or FoxO1 show significant muscle loss and atrophy (Reference S6). Inhibiting FoxO transcriptional activity can reduce Trim63 and Fbxo32 expression, helping to attenuate muscle loss.

SAMP8 is a murine model of accelerated ageing, characterized by a short lifespan and an expedited ageing process. The Senescence Accelerated Mouse Resistant 1 (SAMR1), sharing a common genetic background with SAMP8, serves as the normal control model. In comparison to the SAMR1 mice, SAMP8 mice exhibit a decline in muscle strength, fibre size and phosphocreatine levels in muscles as ageing progresses (Reference S7). SAMP8 mice are the most commonly used accelerated ageing mouse model in the study of sarcopenia [8]. Studies have indicated that the peak muscle mass in SAMP8 mice occurs at 7 months of age; the onset of muscle loss in these animals begins at 8 months, which is mainly manifested as significant loss of muscle mass (12.41%), muscle strength (11.64%) and contractility (25.96%) (Reference S8). The age‐related pathological phenotypes observed during the ageing process of SAMP8 mice are similar to those found in human geriatric diseases. Therefore, SAMP8 is generally recommended as an ideal animal model for sarcopenia research, offering both cost‐effectiveness and high efficiency. In addition, other strains of SAMP mice can also be used in the study of sarcopenia, such as SAMP10 [9], SAMP6 (Reference S9) and SAMP1 (Reference S10).

NTs are important substances in organisms that determine biological characteristics and the structure and function of proteins, control the growth, development, reproduction and heredity of organisms and are metabolic regulators of a variety of nutrients in the body (Reference S11). Dietary nucleotides are considered a conditionally essential nutrient because they tend to be deficient under some conditions [10] (Reference S12). It was previously assumed that NTs were not needed for normal growth and development, but some studies demonstrate that the ability to synthesize NTs from scratch is reduced when the organism is under special circumstances, such as immunological challenges, ageing and stress [10] (Reference S13). Therefore, exogenous acquisition of NTs is a better way to supplement. Exogenous NTs supplementation is beneficial for health, improving immunity (Reference S14), supporting liver function [11] and promoting growth [12]. NTs deficiency, especially in the elderly, can have adverse effects. NTs are reduced in muscles with ageing. Previous studies showed cytidine 5′‐monophosphate, uridine 5′‐monophosphate and adenosine 5′‐diphosphate are reduced in the muscles of old mice [13, 14]. Research indicates that UMP/CMP can increase type II fibre diameters following muscle injury [15]. Our recent study found that 5′‐CMP alleviates H_2_O_2_‐induced muscular atrophy in C2C12 myotubes [16]. These results of studies suggested NTs supplementation could be beneficial for ageing skeletal muscle. Besides, other studies reported that NTs could extend the lifespan of SD rats (Reference S15) and delay the ageing process in cells [17]. Therefore, NTs may be a potential candidate in attenuating age‐related sarcopenia. To evaluate the effect of NTs on sarcopenia, we investigated whether long‐term dietary supplementation with a specific NTs‐enriched mixture could attenuate age‐related sarcopenia in old and very old (geriatric) SAMP8 mice and its possible mechanisms.

## 2 Materials and Methods

### 2.1 Rodent Studies

All murine studies were approved by the Animal Studies Committee of Peking University Health Science Center. We used 130 SPF‐grade male SAMP8 and SAMR1 mice, aged 10–12 weeks, purchased from Peking University's Laboratory Animal Science Department. Mice were housed singly to prevent fighting, maintained at 22 ± 2°C with 50%–60% humidity and kept on a 12‐h/12‐h light/dark cycle. After a 1‐week acclimation, mice were divided into two cohorts. Cohort 1 included six groups (12 mice per group, except 10 in the young control): normal control, three NTs intervention groups, young control and SAMR1. Cohort 2 had five groups (12 mice per group): normal control, three NTs intervention groups and SAMR1. All control and SAMR1 mice received a standard diet (Beijing Keao Xieli Feed Co. Ltd.). NT intervention groups had NTs added to their diet at 0.3, 0.6 and 1.2 g/kg, corresponding to NTs‐low dose group (NTs‐LG), NTs‐medium dose group (NTs‐MG) and NTs‐high dose group (NTs‐HG). The NTs mixture used in vivo experiments was prepared with the following ratio: AMP:CMP:GMP:UMP = 32.6:90.3:33.4:46.8, which is consistent with that found in breast milk and infant formula (Reference S16). Animals were monitored daily for changes in health and behaviour, with weekly recordings of body weight and food intake. Mice from cohort 1 were euthanized at 12 months and those from cohort 2 at 18 months.

### 2.2 Chemicals

5′‐adenosine monophosphate (AMP), 5′‐cytimidine monophosphate (CMP), disodium guanosine 5'‐monophosphate (GMP) and disodium uridine 5'‐monophosphate (UMP) were supplied by Hainan Shuangdi Zhen‐Ao Life Science Research Center Co. Ltd. (Baoting, China).

### 2.3 Cell Culture and Treatments

C2C12 cells were obtained from Zhejiang Meisen Cell Technology Co. Ltd. Details of cell culture and treatments can be found in the Supporting Information.

### 2.4 Analysis of Physical Performance Tests and Body Composition

The physical performance of SAMP8 and SAMR1 mice was assessed through grip strength, wire hang, horizontal bar and gait tests. The methods of grip strength and wire hang have been previously described [18]. For the horizontal bar test, the mice were placed on a custom‐made horizontal bar with a diameter of 10 * 10 mm, and the time they held onto the bar was recorded. We set the cutoff time at 60 s, and the average of two trials was taken as the mice's retention time on the bar. During the gait test, mice were observed in a 60 * 60 * 60 cm box for 5 min, and Smart 3.0 software analysed their movement, including distance and speed. Additionally, body composition, including fat and muscle mass, was measured using EchoMRI following the manufacturer's instructions.

### 2.5 Immunofluorescence Staining and Imaging

In order to perform immunofluorescence staining for muscle sections or C2C12 myotubes, we collected mouse gastrocnemius (GAS) specimens or myotube cells, froze them with liquid nitrogen in tissue‐Tek OCT and then sliced the muscle sections into 20 μm with Cryostat (CM1950, Leica). The specific experimental operation and analytical methods were performed as previously described [16, 18].

### 2.6 RNA‐Seq and Analysis

Following the manufacturer's instructions, total RNA was extracted from quadriceps (QUAD) muscles with TRIzol reagent (TIANGEN, China) and the integrity and total amount of RNA were accurately detected using Agilent 2100 bioanalyzer. RNA‐seq was performed as described previously [16]. The specific experimental operation and analytical methods are also provided in the Supporting Information.

### 2.7 Total RNA Extraction and RT‐qPCR Analysis

Total RNA from tibialis anterior (TA) or myotubes was isolated with TRIzol Reagent (Invitrogen) and quantified with Nanodrop. ReverTra Ace Reverse Transcriptase (TOYOBO) was used to synthesize cDNAs from 1‐μg total RNA following the manufacturer's instructions. RT‐qPCR reactions were performed with SYBR Green (TOYOBO). All data were normalized to normal control group expression. Primer sequences are listed in Table S1.

### 2.8 Western Blot Analysis

The expression of proteins in GAS muscle tissues from mice aged 12 months or myotubes were performed with the following antibodies: Dkk3 (Abcam, ab187532), Mt (Abcam, ab192385), FoxO1 (CST, 2880S), FoxO3a (CST, 2497S), Trim63 (Proteintech, 55456‐1‐AP), Fbxo32 (Abcam, ab168372), phospho‐Akt (thr473) (CST, #4060), Akt (Proteintech, 10 176‐2‐AP), phospho‐S6K (thr389) (CST, #9234), S6K (CST, 9202S), P53 (CST, 2524S) and GADPH (Proteintech, 10494‐1‐AP). The antibodies were listed in Table S2.

### 2.9 Metabolomic Profiles and Data Processing

Metabolomic profiles (*n* = 6/group) were identified using a High Throughput Targeted Quantification Kit (HM350, BGI, Shenzhen, China). Extensor digitorum longus (EDL) muscle tissue was mixed with a 50% water/methanol solution containing an internal standard for quality control. After centrifugation, the supernatant was analysed using LC‐MS/MS on a QTRAP 6500+ (SCIEX, USA). Chromatographic parameters included a BEH C18 column and electron spray ionization (ESI) source. The concentration of metabolites was calculated using the integral peak area and standard curve. Metabolites with a ratio ≥ 1.2 or ≤ 0.83 and an adjusted *p* value < 0.05 were identified as differentially expressed metabolites. Bioinformatics analyses, including KEGG pathway classification, were then performed. The specific experimental operation and analytical methods are also provided in the Supporting Information.

### 2.10 Statistical Analysis

We used SPSS software version 22 (SPSS Inc., Chicago, IL, USA) and R software (v4.4.2)for statistical analyses. All values are expressed as mean ± SEM, unless stated otherwise. Differences between groups were analysed by a one‐way analysis of variance test and LSD methods if the data were homogeneous, or Dunnett's T3 test if variances were unequal. Two‐way repeated‐measure ANOVAs for multiple recordings over time were used to compare between groups with two independent variables. Statistical significance was set at *p* < 0.05. *η* ^2^ evaluates the effect size of NTs intervention by using the following rules of thumb for interpretation of the effect size: Small is 0.01, medium is 0.06 and large is 0.14 [19]. We conducted a Bayesian mediation analysis using structural equation modelling (SEM) to examine the mechanistic pathways linking NTs supplementation to muscle mass changes. We made a mediation analysis in the models, with a decomposition of total effect into direct and indirect effects [20]. The analysis tested two pathways: (1) NTs supplementation → Dkk3 expression → muscle mass; (2) NTs supplementation → Mt1 expression → muscle mass. The gene expression values were log‐transformed to approximate normal distributions. The analysis used Markov chain Monte Carlo (MCMC) sampling with three chains, a burn‐in of 5000 iterations and 30 000 post‐burn‐in samples. A random seed was set (seed = 123) to ensure reproducibility of the results. Posterior means and 95% Bayesian credible intervals (CI) were reported for all path coefficients.

## 3 Results

### 3.1 The Dietary Supplementation of NTs Delays Age‐Related Muscle Mass Wasting in Old and Very Old (Geriatric) SAMP8 Mice

**FIGURE 1:**
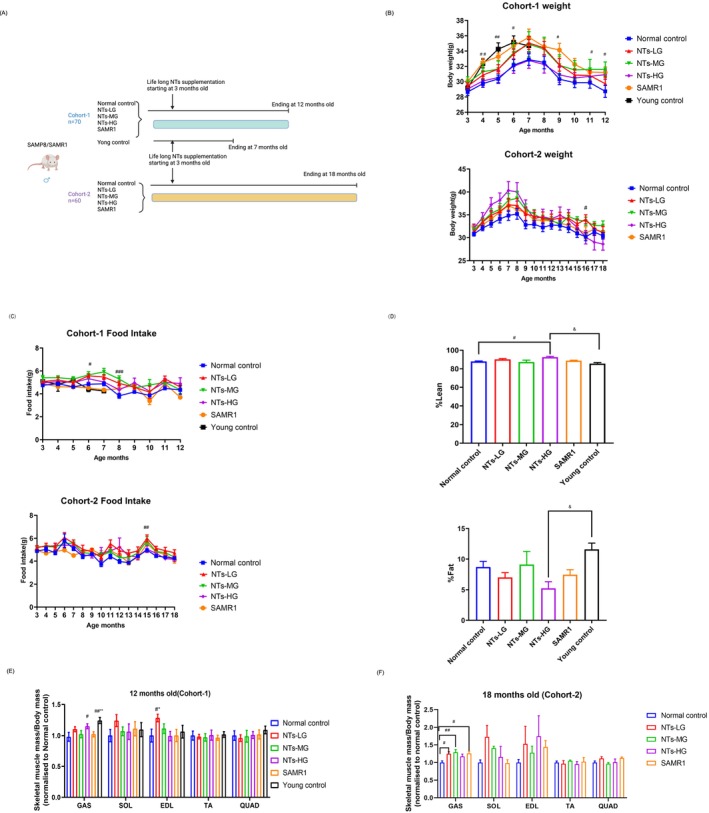
The dietary supplement of NTs delays age‐related muscle mass wasting in old and very old (geriatric) SAMP8 mice. (A) Experimental timeline. Mice were divided into two cohorts consisting of old and geriatric SAMP8 mice (*n* = 12, except the young control group *n* = 10) that were fed NTs for 9 and 15 months, respectively. (B) Body weight for mice receiving NTs intervention or control diet beginning at 3 months and ending until 12 months (Cohort 1; *n* = 12,except Young control group *n* = 10) or 18 months (Cohort 2; *n* = 12). (C) Food intake (g/day) of mice in the two cohorts. (D) Lean/body weight and fat/body weight in each group (*n* = 6). (E) Muscle mass for gastrocnemius (GAS), soleus (SOL), extensor digitorum longus (EDL), tibialis anterior (TA) and quadriceps (QUAD) was normalized to body mass and then to the normal control mice for the 12‐month‐old mice in cohort 1 (*n* = 6–10 mice per group). (F) Muscle mass for GAS, SOL, EDL, TA and QUAD was normalized to body mass and then to the normal control mice for the 18‐month‐old mice in cohort 2 (*n* = 3 mice per group). Error bars show SEM. Two‐way repeated‐measures ANOVAs with Sidak post hoc tests (B–C). One‐way ANOVAs with LSD or Dunnett's T3 (D–F). ^#^ *p* < 0.05, ^##^ *p* < 0.01, ^###^ *p* < 0.001. ^#^ *p* < 0.05 versus the normal control group, *p < 0.05 versus SAMR1, ^&^ *p* < 0.05 versus the young control group.

To evaluate NTs' effects on age‐related muscle decline, we assessed two cohorts of mice, each consisting of 70 males or 60 males mice (totalling 130 mice) (Figure 1A), allowing us to assess reproducibility for sarcopenia effects of NTs and analyse the effect of NTs on muscle properties in SAMP8 mice of 7 (young), 12 (old) and 18 (geriatric) months of age. Ageing signs in SAMP8 mice appear at 8 months, including rough, thinning hair, and by 10–18 months, symptoms like yellowing hair, hair loss, ulcers, spinal protrusion and slow gait become evident. NTs improved ageing status to some extent.

Research shows SAMP8 mice reach peak muscle mass at 7 months, with significant declines in muscle mass (12.41%), strength (11.64%) and contractility (25.96%) starting at 8 months (Reference S8). Our study confirmed these findings: muscle mass peaks at 7 months and declines by 8 months (Figure 1B). The results of variance analysis of repeated measurements showed that time had a certain effect on the weight of mice. With the passage of time, the body mass of mice showed a certain trend of change over time: The body mass of mice in both cohorts gradually increased at 3–7 months of age, reached the maximum weight at 7 months of age, which is consistent with the results of Guo et al. (Reference S8), and after 7 months, the body mass began to decline until about 10 months of age, at which point body mass slowly declined. The body mass of old mice in the NTs‐MG, where they were in cohort 1, was significantly heavier than normal control mice at 11 months. The body mass of very old (geriatric) mice in the NTs‐LG from cohort 2 was significantly heavier than normal control mice at 16 months of age. The results indicated that NTs could increase the body mass of old and geriatric mice with sarcopenia. Food intake results showed no significant differences among the groups for most months, with the exception of the following months: the 6‐ and 8‐month‐old mice in the NTs‐LG of cohort 1, as well as the 8‐month‐old mice in the NTs‐MG, consumed more food than the normal control group. In cohort 2, the 15‐month‐old mice in the NTs‐LG group also exhibited higher food intake compared with the control group (Figure 1C). In addition, we compared differences in body weight and food intake between the two cohorts of mice. The results showed that from 3 to 12 months of age, mice in cohort 2 exhibited consistently higher body weights than those in cohort 1 (Figure S1). Regarding food intake, mice in cohort 2 consumed significantly more food at 5, 6 and 7 months of age compared with cohort 1. At other time points, no statistically significant differences in food intake were observed between the two cohorts (Figure S2).

To determine the effect of NTs on body composition, we measured body composition by EchoMRI analysis meter. Compared with the normal control group, lean mass increased significantly in the NTs‐MG, NTs‐HG, SAMR1 and young control group (Figure S3). Furthermore, lean mass/body weight increased significantly in the NTs‐HG group (*p* < 0.01, *η* ^2^ = 0.434) (Figure 1D). Compared with the young control group, the fat/body weight of mice in the NTs‐HG group was significantly decreased (*p* < 0.05, *η* ^2^ = 0.3443) (Figure 1D). The result indicated that NTs supplementation could increase lean mass without raising fat mass. The GAS muscle mass of mice was significantly lower in the normal controls group compared with the young control group. NTs supplementation mitigated the age‐related loss of relative muscle mass in the GAS and EDL muscles in cohort 1 (*p* < 0.05, *η* ^2^ = 0.256；*p* < 0.05, *η* ^2^ = 0.158) (Figure 1E). NTs also increased relative muscle mass in the GAS of geriatric mice in cohort 2 (*p* < 0.05, *η* ^2^ = 0.587) (Figure 1F).

### 3.2 The Dietary Supplementation of NTs Increases the CSA of Myofibers

**FIGURE 2:**
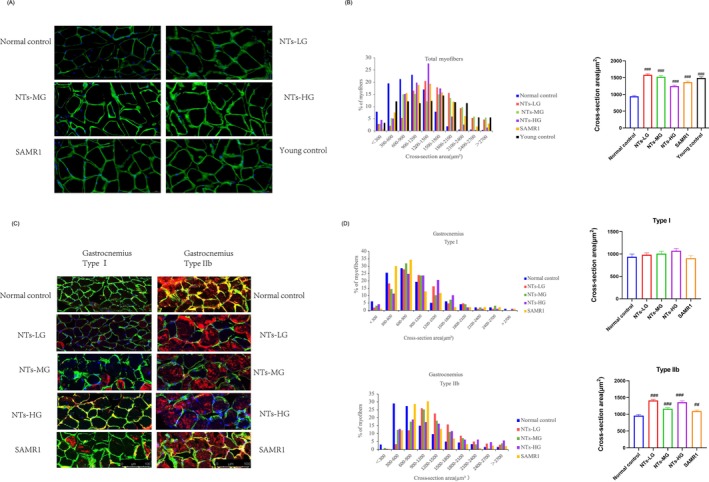
The dietary supplementation of NTs increases the cross‐sectional area (CSA) of myofibers. (A) Immunofluorescent staining images of muscle cross sections derived from gastrocnemius （GAS） of old mice in cohort 1. Green indicated laminin staining. Scale bars, 20 μm. (B) Percentage distribution of muscle fiber CSA (left) derived from GAS (400 < *n* < 500) and were based on three independent experiments, box plot representing the mean CSA (right) of muscle fibers. C‐D Effects of NTs on MyHC expression in geriatric mice from cohort 2 (*n* = 3). (C) Representative images and quantification of laminin (green), MyHC I (red), MyHC IIb (red) immunofluorescent staining in GAS (*n* = 3). (D) Percentage distribution of CSA for MyHC I and MyHC IIb muscle fibers  derived from GAS and the mean CSA of muscle fibers.

To examine NTs' effects on myofiber size, we performed immunostaining on GAS muscle from both cohorts. Laminin staining of GAS muscle cross‐sections showed that NTs increased the CSA of muscle fibers in old mice (*p* < 0.0001, *η* ^2^ = 0.1081) (Figure 2A,B), with a notable increase in larger fibers. Additionally, staining for type IIb myosin heavy chain (MHC) in 18‐month‐old SAMP8 mice revealed marked atrophy of muscle fibers compared with SAMR1 mice (Figure 2C,D). The proportion of coarse type II muscle fibers of GAS in the NTs intervention group was significantly higher than that in the normal control group. The CSA of type II muscle fibers in the NTs intervention group was larger than that in the normal control group (*p* < 0.0001, *η* ^2^ = 0.0699). However, no significant differences were observed in the CSA of type I muscle fibers across the groups. These results suggest that NTs intervention increases the CSA of muscle fibers and the proportion of coarse myofibers, particularly enhancing type IIb fibers, which are highly sensitive to age‐induced apoptosis [21].

### 3.3 The Dietary Supplementation of NTs Improves Physical Performance, Reduces Muscle Fibrosis and Inflammation in old SAMP8 Mice

**FIGURE 3:**
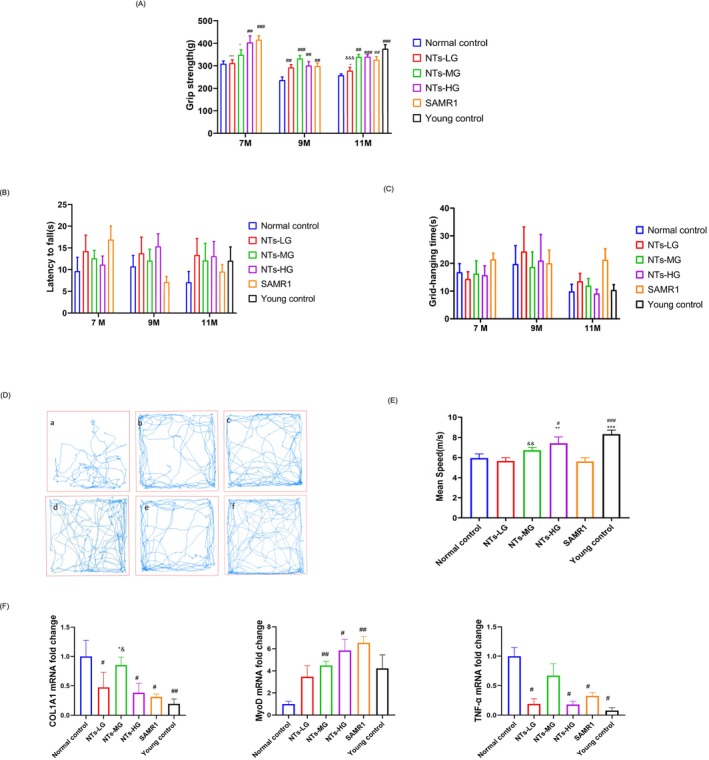
The dietary supplementation of NTs improves physical performance in old SAMP8 mice. (A) Grip strength test, (B,C) grid‐hanging capacity, (B) horizontal bar test, (C) wire hang test, (A–C, *n* = 10 mice per group in 11 months age), (D) movement track of 11‐month‐old mice in 5 min. (E) Mean speed (*n* = 10 mice per group in 11 months age). (F) mRNA expression of COL1A1, MyoD and TNF‐α (*n* = 6 mice per group). Expression is shown relative to that of the normal control group. Error bars show SEM. One‐way ANOVAs with LSD or Dunnett's T3. ^#^ *p* < 0.05, ^##^ *p* < 0.01, ^###^ *p* < 0.001. ^#^ *p* < 0.05 versus the normal control group, *p < 0.05 versus SAMR1, ^&^ *p* < 0.05 versus the young control group.

To further investigate the effects of NTs on physical performance, we assessed the physical performance of mice in cohort 1. We found that NTs significantly increased muscle grip strength at 7 (*p* < 0.0001, *η* ^2^ = 0.312), 9 (*p* < 0.001, *η* ^2^ = 0.293) and 11 (*p* < 0.0001, *η* ^2^ = 0.507) months of age (Figure 3A). However, there were no significant differences between groups in the horizontal bar test or wire hang test (Figure 3B,C). At 11 months, the mice underwent a gait test, which revealed that the gait speed in the NTs‐HG group was significantly increased compared with the normal control group (*p* < 0.0001, *η* ^2^ = 0.3861) (Figure 3D,E). The SAMP8 mice in cohort 2 were used to evaluate the effects of NTs on sarcopenia in geriatric mice. To avoid any influence of behavioural experiments on the geriatric mice's lifespan or health status, no behavioural experiments were conducted in cohort 2. To further evaluate the effects of NTs supplementation on muscle fibrosis, inflammatory cytokines and muscle stem cells in aged mice, we used RT‐qPCR to detect the mRNA levels of COL1A1, TNF‐α and MyoD. The results showed that NTs supplementation significantly reduced the mRNA levels of COL1A1 (*p* < 0.05, *η* ^2^ = 0.3417) and TNF‐α (*p* < 0.0001, *η* ^2^ = 0.6135), while increasing the level of MyoD (*p* < 0.01, *η* ^2^ = 0.4803) (Figure 3F).

### 3.4 NTs Protects Age‐Related Muscle Atrophy by Inhibiting Foxo, P53 Pathway and Activating mTOR Pathway in SAMP8 Mice and C2C12 Cells

**FIGURE 4:**
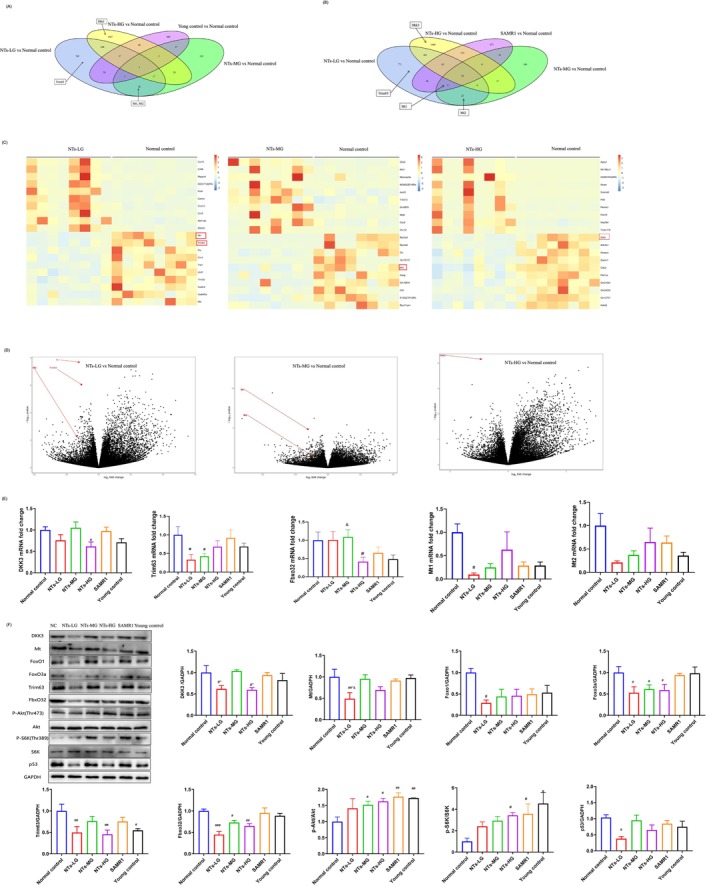
NTs protects age‐related muscle atrophy by inhibiting of Foxo,P53 and activating mTOR signalling pathways in SAMP8 mice. (A,B) Venn diagram of the overlap among significantly upregulated or downregulated genes in NTs‐LG, NTs‐MG, NTs‐HG, and the young control group compared with the normal control group and NTs‐LG, NTs‐MG, NTs‐HG and SAMR1 compared with the normal control group (|log2foldchange| > 1 and *p* < 0.05). (C) Heatmap of upregulated and downregulated Top10 genes in NTs‐LG versus the normal control group, NTs‐MG versus the normal control group, NTs‐HG versus the normal control group. (D) Volcano plot showing differentially expressed genes (DEG) in NTs‐LG, NTs‐MG and NTs‐HG, compared with the normal control group. (E) Tibialis anterior mRNA expression of genes involved in sarcopenia that were screened by RNA‐seq (*n* = 6 mice per group). Expression is shown relative to that of the normal control group. (F) Protein expression of genes where are in Foxo, Akt/mTOR and p53 pathways involved in sarcopenia (*n* = 3 mice per group). Error bars show SEM. One‐way ANOVAs with LSD or Dunnett's T3 (D‐G). ^#^ *p* < 0.05, ^##^ *p* < 0.01, ^###^ *p* < 0.001. ^#^ *p* < 0.05 versus the normal control group, *p < 0.05 versus SAMR1, ^&^ *p* < 0.05 versus the young control group.

To assess the impact of NTs supplementation on gene expression in the muscles of aged SAMP8 mice, we analysed QUAD muscles from 7‐month‐old (young) and 12‐month‐old (old) SAMP8 mice, as well as 12‐month‐old SAMR1 mice. RNA‐seq was performed on these muscle samples, and genes with log2foldchange > 1 or < −1 and *p* < 0.05 were identified as differentially expressed. The Venn diagram of significantly differentially expressed genes in NTs‐LG, MG, HG and young control groups versus the normal control group revealed 768 shared genes in NTs‐LG versus normal control, including Trim63 (MuRF1), a key marker of muscle atrophy, which was significantly downregulated (Reference S4) (Figure 4A). The overlap of NTs‐LG versus normal control and NTs‐MG versus normal control identified 26 differentially expressed genes, notably downregulated Mt1 and Mt2. Previous studies have reported that metallothioneins in models of rodent and human skeletal muscle atrophy displayed a significant increase [22, 23] (References S17 and S18) and genetic silencing of these genes promotes muscle hypertrophy in vivo [22]. NTs‐HG versus normal control, 1507 genes were differentially expressed, with Dkk3 significantly downregulated. Dkk3 is a marker for sarcopenia and its expression increases with age‐related muscle atrophy [24]. The Venn diagram of NTs‐LG, MG, HG and SAMR1 groups versus normal control identified four shared genes among NTs‐LG, MG and SAMR1, including Mt1 (Figure 4B). Heatmap and volcano plot analyses showed significant decreases in Mt1 and Trim63 expression in NTs‐LG versus normal control, with these genes ranking among the top two downregulated in muscle (Figure 4C,D). NTs‐MG versus normal control also showed significant decreases in Mt1, which was among the top five downregulated genes (Figure 4C,D). For NTs‐HG versus normal control, Dkk3 was the top one downregulated gene (Figure 4C,D).

To validate RNA‐seq results, we examined the sarcopenia‐related genes at both mRNA and protein levels. RT‐qPCR confirmed the downregulation of Fbxo32 (*p* < 0.05, *η* ^2^ = 0.3861), Trim63 (*p* < 0.05, *η* ^2^ = 0.3094), Dkk3 (*p* < 0.05, *η* ^2^ = 0.3188) and Mt1 (*p* < 0.05, *η* ^2^ = 0.3576) in the NTs intervention group (Figure 4E). Mt2 showed a downward trend but was not statistically significant (Figure 4E). Additionally, NTs decreased protein expression of Fbxo32, Trim23, Dkk3, Mt, Foxo1 and FoxO3a, while increasing Akt and P70 S6 kinase (P70S6K) phosphorylation, which are related to protein synthesis and degradation (Figure 4F). Because Dkk3 is a secreted protein, we investigated whether Dkk3 protein levels in circulation were also downregulated in the NTs intervention groups using ELISA. The serum Dkk3 concentration was significantly lower in NTs intervention groups (Figure S4). Western blot analysis revealed a significant decrease in the age‐related marker p53 in muscles of the NTs intervention group compared with controls. These findings suggest that NTs inhibit protein degradation and promote protein synthesis by modulating the Foxo and mTOR signalling pathways, improving age‐related skeletal muscle ageing.

To verify the effects of nucleotide monomers and mixtures on muscle atrophy, we investigated their impact on H_2_O_2_‐induced C2C12 muscle atrophy cells using different concentrations of AMP, CMP, GMP, UMP and a mixture of nucleotides (Figure S5A). The CCK‐8 assay showed that 100μM H_2_O_2_ significantly decreased cell proliferation in C2C12 cells, consistent with prior findings that H_2_O_2_ induces muscle atrophy [25]. In the subsequent experiments, we used 100μM H_2_O_2_ to induce muscle atrophy. The CCK‐8 assay demonstrated that NTs improved cell viability in C2C12 myotubes (Figure S6). Myotube diameter was assessed via immunofluorescence staining. The diameter was significantly reduced in the model group but increased in all NTs treatment groups, indicating that AMP, CMP, GMP, UMP and their mixture mitigated myotubes atrophy (Figure S5B,C). NTs treatment also significantly reduced the mRNA levels of Fbxo32 (*p* < 0.05, *η* ^2^ = 0.5681), Trim23 (*p* < 0.05, *η* ^2^ = 0.5957), Dkk3 (*p* < 0.001, *η* ^2^ = 0.8267), Mt1 (*p* < 0.05, *η* ^2^ = 0.5503) and Mt2 (*p* < 0.05, *η* ^2^ = 0.5625) in C2C12 myotubes (Figures S5D). To evaluate NTs' impact on the Akt/Foxo signalling pathway, we detected sarcopenia‐related proteins. Results indicated that NTs targeted the Foxo signalling pathway and influenced mTOR and P70S6K phosphorylation, affecting muscle catabolic and anabolic processes during ageing. Notably, NTs increased Akt and S6K phosphorylation and reduced Trim63 and Fbxo32 protein expression (Figures S5E). Consistent with animal experiments, western blotting showed a significant decrease in the age‐related marker p53 in AMP, CMP, GMP, UMP and mixture groups compared with the model group (Figures S5E). Moreover, the effects of the nucleotide mixture seemed to be more pronounced in the mRNA and protein expression of genes related to sarcopenia.

### 3.5 Bayesian Mediation Analysis Identifies Dkk3 as a Potential Mediator of Muscle Mass

To further investigate whether NTs intervention affects muscle mass through the mediators Mt1 and Dkk3, we conducted a Bayesian mediation analysis. Bayesian mediation analysis identified Dkk3 as a statistically supported mediator linking NTs supplementation to muscle mass enhancement, with strong evidence for a full mediation effect observed in the high‐dose group. In the NTs‐HG versus the normal control group, the NTs‐HG was associated with a significant downregulation of Dkk3 expression (95% CI: −1.659 to −0.326), while Dkk3 expression was negatively associated with muscle mass (95% CI: −4.644 to −2.339). The indirect effect was significantly positive (indirect = 3.473, 95% CI: 0.879–6.067), whereas the direct effect was not statistically significant (direct = 0.237, 95% CI: −1.717 to 2.192), suggesting that Dkk3 fully mediated the relationship between NTs supplementation and muscle mass. The total effect was also significant (total = 3.710, 95% CI: 0.883–6.537). No statistically significant mediation effect was observed for Mt1 in the models tested. Although Mt1 was not identified as a statistically significant mediator in the Bayesian mediation analysis, its expression was markedly downregulated following NTs supplementation, at both the mRNA and protein levels. These findings, supported by previous mechanistic studies, warranted further investigation into the upstream regulatory mechanisms and metabolic associations of Mt1 and Dkk3.

### 3.6 NTs Changed Metabolomic Profiles in the Skeletal Muscle of SAMP8 Mice

**FIGURE 5:**
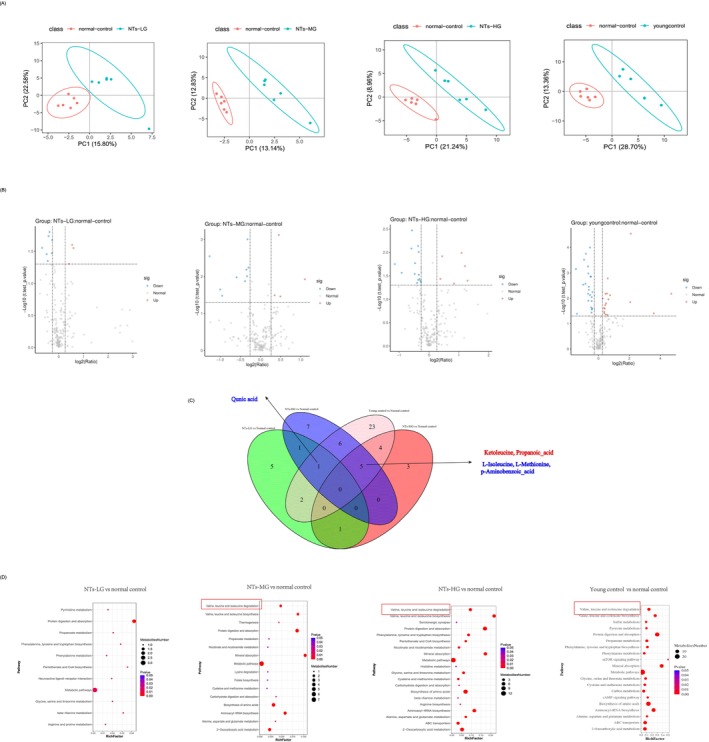
NTs changed metabolomic profiles in the skeletal muscle of SAMP8 mice. (A) PLS‐DA score plot in NTs‐LG, NTs‐MG, NTs‐HG and the young control group versus the normal control group, respectively. PC1 and PC2 represent the scores of the first and second principal components, respectively. (B) Volcano plot showing differentially expressed metabolites in NTs‐LG, NTs‐MG, NTs‐HG and the young control group, compared with the normal control group. (C) Venn diagram of the overlap among significantly upregulated or downregulated differential metabolites in NTs‐LG, NTs‐MG, NTs‐HG and the young control group compared with the normal control group. (D) The enriched top 1–20 KEGG pathways sorted by *p* value based on the differential metabolites of NTs‐treated groups, the young control group versus the normal control group. The size of the point indicates the number of enriched metabolites (*n* = 6 mice per group).

There is no research on muscle metabolites in young and old SAMP8 mice. To characterize metabolic changes in skeletal muscle during ageing, we explore metabolic changes in skeletal muscle during ageing in SAMP8 mice, focusing on the effects of NTs. Using HM350‐targeted metabolomics analysis on the EDL muscle of young (7 months) and old (12 months) mice, a distinct distribution of metabolites was observed between these groups (Figure 5A). A total of 10, 13 and 20 identified metabolites were found in the NTs‐LG, NTs‐MG and NTs‐HG groups compared with the normal control, respectively (Figure 5B); 18 metabolites, such as ketoleucine, 3‐hydroxylisovalerylcarnitine, 3‐methyl‐2‐oxovaleric acid and carnosine, were significantly upregulated in young mice (Table S3), while 23 metabolites, including amino acids like l‐alanine, l‐asparagine and l‐methionine, were downregulated (Table S3). These findings provide new insights into metabolic changes in the muscles of SAMP8 mice with sarcopenia. NTs can shift the muscle metabolic profile of aged mice towards that of young mice. NTs supplementation significantly increased metabolites like ketoleucine, carnitine, 3‐hydroxylisovalerylcarnitine and 3‐methyl‐2‐oxovaleric acid while decreasing others such as l‐alanine, l‐asparagine and l‐methionine, which were similar to those of the young control group (Table S4). The Venn diagram indicated that ketoleucine and propanoic acid were co‐upregulated among the NTs‐MG, NTs‐HG and young control groups compared with the normal control, while co‐downregulated metabolites included l‐isoleucine, l‐methionine and *p*‐aminobenzoic acid (Figure 5C, Figure S7). KEGG pathway analysis indicated that NTs supplementation primarily affected amino acid metabolic pathways, including those related to protein digestion and the metabolism of valine, leucine, isoleucine, glycine, serine, threonine and methionine (Figure 5D). These pathways are associated with sarcopenia‐related traits and muscle regeneration [26]. Among them, the valine, leucine and isoleucine degradation pathway was the top 1 pathway, which was affected in the young control group versus normal control group, NTs‐MG versus normal control group and NTs‐HG versus the normal control group (Figure 5D). Among the upregulated metabolites, ketoleucine, 3‐methyl‐2‐oxovaleric acid and 3‐hydroxylisovalerylcarnitine are metabolites generated via the catabolism of branched‐chain amino acids (BCAAs). Ketoleucine, 3‐methyl‐2‐oxovaleric acid and 3‐hydroxylisovalerylcarnitine are important intermediates in leucine and isoleucine catabolism [27]. This suggests that NTs may potentially affect the valine, leucine and isoleucine pathway, leading to an elevation in the levels of metabolites, for example, ketoleucine, 3‐methyl‐2‐oxovaleric acid and 3‐hydroxylisovalerylcarnitine.

### 3.7 Integrated Analyses of RNA‐Seq and Metabolomic in the Skeletal Muscle of SAMP8 Mice

**FIGURE 6:**
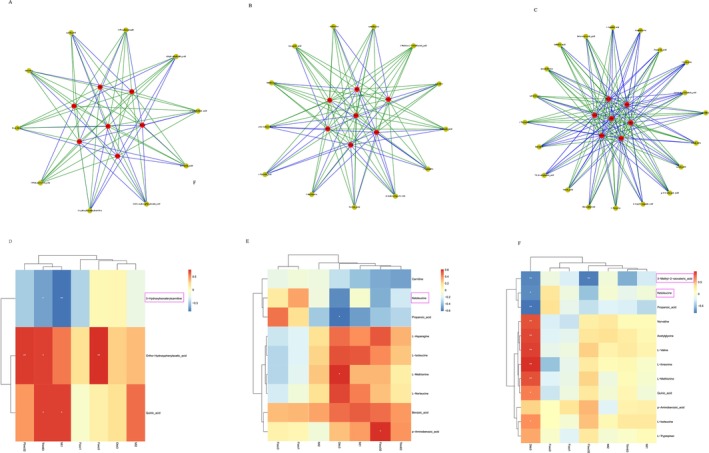
Integrated analyses of RNA‐seq and Metabolomic in the skeletal muscle of SAMP8 mice. (A–C) Network analysis of differential metabolites and target genes in the NTs‐LG, NTs‐MG and NTs‐HG versus the normal control group, respectively. (D) Heatmap of common differential metabolites and target genes from the NTs‐LG versus normal control group and the young control group versus the normal control group and target genes. (E) Heatmap of common differential metabolites from the NTs‐MG versus the normal control group and the young control group versus the normal control group and target genes. (F) Heatmap of common differential metabolites from the NTs‐HG versus the normal control group and the young control group versus the normal control group and target genes (*n* = 6).

To explore the relationship between target genes and differential metabolites, integrated RNA‐seq and metabolomics analyses were performed. In the NTs‐LG group versus the normal control group, 2‐methylhexanoic acid was positively correlated with Trim63, Mt1 and Mt2 (Figure 6A). Mt2 was also linked to isocaproic acid, beta‐alanine and ethylmethylacetic acid. In the NTs‐MG group, metabolites such as isocaproic acid, l‐isoleucine and l‐methionine were positively correlated with Mt1 and Mt2 (Figure 6B). In the NTs‐HG group, valeric acid, l‐isoleucine, ethylmethylacetic acid and *p*‐aminobenzoic acid were strongly positively correlated with Dkk3, whereas l‐tryptophan, l‐methionine, l‐valine, l‐threonine and acetylglycine were negatively correlated with Mt1 and Mt2 (Figure 6C). Additionally, 5‐aminolevulinic acid was positively correlated with Foxo3, and guanosine monophosphate was negatively correlated with Foxo3 in the young versus the normal control group (Figure S8). To evaluate the metabolite reversal of old mice to young mice after NTs supplementation and their relationship with targeted genes, we examined the overlap between NTs supplementation and young control groups concerning differential metabolites and the association with targeted genes. The differential metabolites and targeted genes with *R* > 0.6 and *p* < 0.05 were selected for visualization. Ortho‐hydroxyphenylacetic acid was significantly positively correlated with Fbxo32 and Trim63. Additionally, 3‐hydroxylisovalerylcarnitine was significantly negatively correlated with Mt1 (Figure 6D). l‐methionine was positively correlated with Dkk3, while *p*‐aminobenzoic acid was negatively correlated with Fbxo32 (Figure 6E). In the NTs‐HG group, ketoleucine, 3‐methyl‐2‐oxovaleric acid and propanoic acid were negatively correlated with Dkk3, while acetylglycine, l‐isoleucine and quinic acid were positively related to Dkk3 (Figure 6F). Therefore, through correlation analysis, we identified the relationships between ketoleucine, 3‐methyl‐2‐oxovaleric acid and 3‐hydroxylisovalerylcarnitine with the target genes. Ketoleucine and 3‐methyl‐2‐oxovaleric acid are negatively correlated with Dkk3, while 3‐hydroxylisovalerylcarnitine is negatively correlated with Mt1.

## 4 Discussion

Age‐related sarcopenia and muscle strength loss are major causes of disability in the elderly, leading to conditions like congestive heart failure, cognitive decline and osteoporosis [1]. Preventive strategies are crucial for mitigating sarcopenia and preventing disability among older individuals. This study shows that long‐term NTs supplementation, starting at 3 months of age, reduces muscle mass loss in old and geriatric SAMP8 mice. In vitro experiments confirmed that nucleotide monomers—AMP, CMP, GMP and UMP—and their mixtures increased muscle cell diameter and reduced sarcopenia‐related gene expression, aligning with recent studies on CMP and UMP [28, 29]. These findings suggest that NT‐enriched mixtures and their monomers are potential targets for preventing and treating sarcopenia.

Sarcopenia mainly manifests as a reduction in muscle mass, strength and physical performance. Our findings showed that both old and very old (geriatric) SAMP8 mice exhibited clear signs of sarcopenia. NTs supplementation increased body mass, particularly lean body mass, without increasing fat mass. Long‐term NTs supplementation also increased the wet weight of the GAS and EDL muscle. Sarcopenia involves a reduction in myofiber number and size, loss of motor units and changes in fibre‐type composition [30]. NTs supplementation significantly increased the CSA of muscle fibers in old mice and improved CSA and type IIb fibers in geriatric mice, with minimal effects on type I fibers. These results suggest that NTs improve muscle size by increasing IIb muscle fibers.

Manifestations to further investigate the effects of NTs on physical performance, we evaluated grip strength, horizontal bar test, wire hang test and gait speed in old mice. Handgrip strength and gait speed are recommended as effective tests for assessing the physical manifestations of sarcopenia. NTs supplementation improved grip strength at 7, 9 and 11 months and gait speed at 11 months. These results indicate that NTs significantly alleviate physical performance associated with sarcopenia in ageing mice. In addition, NTs supplementation could also reduce muscle fibrosis, inflammation level and increase the level of MyoD.

The molecular mechanisms of sarcopenia remain under exploration. The UPS is key for muscle protein balance. Ageing increases Fbxo32 and Trim63 levels in the UPS, enhancing muscle loss. Akt can suppress this process by inhibiting Foxo transcription factors, crucial for activating atrophy‐related ligases like Fbxo32 and Trim63, leading to muscle loss [7, 31]. Our RNA‐seq results showed that NTs‐LG supplementation downregulated Trim63 transcription. Both Trim63 and Fbxo32 mRNA and protein levels decreased, as did Foxo1 and Foxo3 protein levels, and p‐Akt/Akt and p‐p70/p70 ratios increased. Metallothioneins (Mt), small cysteine‐rich proteins, are linked to intracellular zinc storage and transport. Mt1 is upregulated in atrophying muscle [32] (Reference S19). Mt is important for zinc buffering, which can activate pathways like mTOR [33]. Silencing Mt1 and Mt2 activates the Akt/mTOR pathway, increasing myotube size in vitro [22]. Our findings align with previous reports, showing significant downregulation of Mt1 in the NTs‐LG and NTs‐MG groups. Dkk3 is a glycoprotein that aggravates age‐related muscle atrophy by increasing nuclear β‐catenin import and its interaction with FoxO3, activating Fbxo32 and Trim63 transcription [24] (Reference S20). In addition, overexpression of Dkk3 inhibits the AKT/mTOR pathway, promoting muscle atrophy [26, 34] (Reference S21). Our study showed NTs significantly decreased Dkk3 expression in the NTs‐HG group. To further clarify the mechanistic pathways by which NTs exert their protective effects on muscle mass, we performed a Bayesian mediation analysis to evaluate whether Mt1 and Dkk3 acted as molecular mediators linking NTs supplementation to muscle phenotype improvements. Interestingly, Dkk3 was identified as a statistically significant and complete mediator in the high‐dose group, suggesting that downregulation of Dkk3 may be a key mechanism underlying NTs‐induced muscle protection. However, Mt1 did not meet the criteria for statistical mediation despite its consistent downregulation at both transcript and protein levels. This discrepancy may be attributable to regulatory complexity, timing or nonlinear pathways and prompted us to further investigate its upstream metabolic regulation. Consistent with the results of animal experiments, in vitro studies further confirmed that AMP, CMP, GMP, UMP and their mixture increased C2C12 cell diameter and decreased expression of sarcopenia‐related genes.

The KEGG analysis of targeted metabolomics revealed that NTs also significantly affected protein digestion, absorption and amino acid metabolism. NTs supplementation increased levels of carnitine and ketoleucine (alpha‐ketoisocaproic acid, KIC), both of which are crucial for muscle energy metabolism. Carnitine deficiency is linked to sarcopenia [35], and its supplementation can increase lean mass in cancer patients [36]. Similarly, KIC is known to enhance muscle protein synthesis [37] (Reference S22). KIC could promote protein synthesis by activating mTORC1 signalling [38]. Metabolomic profiling of skeletal muscle in aged FBN rats revealed that increased levels of amino acids were related to muscle loss [13]. As a result of promoted muscle proteolysis, free amino acids were largely released [1] (Reference S23). Consistently, our study showed NTs supplementation significantly decreased these amino acids levels, for example, l‐alanine, l‐isoleucine and l‐methionine. Moreover, our studies reported NTs supplementation could reverse muscle metabolites in old mice to young mice. Integrated RNA‐seq and metabolomic analyses further showed that NTs could cause changes in metabolites by affecting valine, leucine and isoleucine degradation pathways, such as ketoleucine, 3‐hydroxylisovalerylcarnitine and 3‐methyl‐2‐oxovaleric acid, which could further regulate sarcopenia‐related genes (Dkk3, Mt1) and inhibit muscle protein degradation and promote muscle protein synthesis.

**FIGURE 7:**
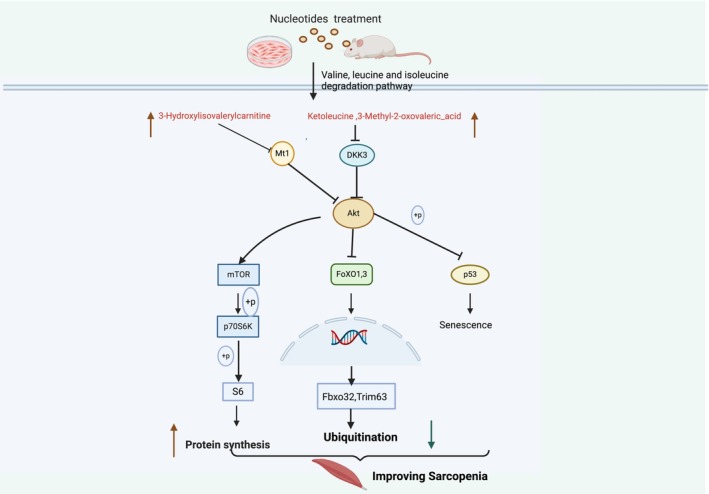
Schematic illustration summarizing the mechanisms by which NTs supplementation affects metabolites such as ketoleucine, 3‐Hydroxylisovalerylcarnitine, 3‐Methyl‐2‐oxovaleric_acid, downregulates Dkk3 and Mt1genes, further inhibits the Foxo signalling pathway, decreases Trim63 and Fbxo32 expression mainly and activates the mTOR/S6K pathway as well as inhibiting the p53 signalling pathway, thus reducing protein degradation, promoting muscle protein synthesis and improved sarcopenia.

In conclusion, our study on NTs long‐term supplementation supports its effectiveness as an intervention for sarcopenia, both in vitro and in vivo. The specific mechanism may be associated with NTs increasing metabolites such as ketoleucine, 3‐hydroxylisovalerylcarnitine and 3‐methyl‐2‐oxovaleric acid by affecting the valine, leucine and isoleucine degradation pathways, downregulating Dkk3 and Mt1 genes, which further decrease protein degradation by inhibiting the Foxo signalling pathway and promoting protein synthesis by activating the Akt/mTOR signalling pathway as well as relieving ageing by inhibiting the p53 signalling pathway (Figure 7). Our study is the first to demonstrate the beneficial effects of NT supplementation on sarcopenia in both old and geriatric SAMP8 mice. A limitation of this study is the lack of direct assessment of protein synthesis by the SunSET assay. Further research is needed to determine if NTs supplements are effective in humans. In addition, the absorption and metabolism of NTs in vivo after consumption also need to be further investigated.

## Conflicts of Interest

The authors declare no conflicts of interest.

## Supporting information


**Figure S1.** Effects of NTs supplementation on body weight in two cohorts. ^###^ *p* < 0.001. ^###^p < 0.001 Cohort‐2 versus Cohort‐1.
**Figure S2**. Effects of NTs supplementation on food intake(g/day) in two cohorts. ^#^ *p* < 0.05, ^##^ *p* < 0.01**.** ^#^ *p* < 0.05, ^##^p < 0.01 Cohort‐2 versus Cohort‐1. 
**Figure S3.**The effect of NTs supplementation on lean mass in SAMP8 mice. Error bars indicated SEM. one‐way ANOVAs with LSD or Dunnetts’T3. ^#^p < 0.05, ^###^ *p* < 0.001. ^#^p < 0.05 versus Normal control group. ^&&^ *p* < 0.01 versus Young control group.
**Figure S4.** The effect of NTs supplementation on serum Dkk3 concentration in SAMP8 mice. Error bars indicated SEM. one‐way ANOVAs with LSD or Dunnetts’T3. ^#^ *p* < 0.05, ^##^p < 0.01, ^###^ *p* < 0.001. ^#^p < 0.05 versus Normal control group.
**Figure S5.** NTs ameliorates muscular atrophy in cultured C2C12 cells. A. Experimental timeline of C2C12 cells. B. Impact of NTs mixture 100, AMP 100, CMP 100, GMP 100 and UMP 100 on the myotube atrophy in C2C12 myotube(*n* = 3. B . Representative images of myotubes. Scale bar = 100 μm. Green indicated Desmin staining, blue indicated DAPI staining of nuclei. C. Average diameters of myotubes(*n* = 3). D. mRNA expression of genes involved in muscular atrophy those are consistent with animal experiment (*n* = 3). Expression is shown relative to that of Model group. E. Protein expression of genes where are in Foxo, Akt/mTOR, and p53 pathways involved in sarcopenia which are consistent with animal experiment (n = 3). C‐D. Error bars indicated SEM. one‐way ANOVAs with LSD or Dunnetts’T3. ^#^ *p* < 0.05, ^##^ *p* < 0.01, ^###^ *p* < 0.001. ^#^ *p* < 0.05 versus Model group.
**Figure S6.** The effect of NTs supplementation on the cell viability in C2C12 muscle atrophy cells. Error bars indicated SEM. one‐way ANOVAs with LSD or Dunnetts’T3. ^#^p < 0.05, ^##^p < 0.01, ^###^p < 0.001. ^#^p < 0.05 versus Model group.
**Figure S7.** a‐f Quantitative analysis chart of common differential metabolites in different NTs‐treated groups vs Normal control group and Young control group vs Normal control group such as Ketoleucine propanoic_acid, L‐Isoleucine, L‐Methionine, p‐Aminobenzoic_acid and Qunic_acid in different groups.
**Figure S8.** Network analysis of differential metabolites and target genes in the Young control group vs Normal control group.
**Table S1.** Real‐time qPCR primer sequences in this study.
**Table S2.** Antibody.
**Table S3.** The differential metabolites in the skeletal muscle of SAMP8 mice in the Young control group vs normal control group.
**Table S4.** The differential metabolites in the skeletal muscle of SAMP8 mice in the NTs supplementation group vs normal control group.

**Data S1.** Supplementary Information.
