# Supplementary material for: Exogenous Nucleotides Supplementation Attenuates Age‐Related Sarcopenia
Source: J Cachexia Sarcopenia Muscle. 2025 Jul 31;16(4):e70002. doi: 10.1002/jcsm.70002 (PMC12313545; doi:10.1002/jcsm.70002)
Supplement: Supplementary file 1 — Figure S1. Effects of NTs supplementation on body weight in two cohorts. ### p < 0.001. ###p < 0.001 Cohort‐2 versus Cohort‐1. Figure S2. Effects of NTs supplementation on food intake(g/day) in two cohorts. # p < 0.05, ## p < 0.01. # p < 0.05, ##p < 0.01 Cohort‐2 versus Cohort‐1. Figure S3.The effect of NTs supplementation on lean mass in SAMP8 mice. Error bars indicated SEM. one‐way ANOVAs with LSD or Dunnetts’T3. #p < 0.05, ### p < 0.001. #p < 0.05 versus Normal control group. && p < 0.01 versus Young control group. Figure S4. The effect of NTs supplementation on serum Dkk3 concentration in SAMP8 mice. Error bars indicated SEM. one‐way ANOVAs with LSD or Dunnetts’T3. # p < 0.05, ##p < 0.01, ### p < 0.001. #p < 0.05 versus Normal control group. Figure S5. NTs ameliorates muscular atrophy in cultured C2C12 cells. A. Experimental timeline of C2C12 cells. B. Impact of NTs mixture 100, AMP 100, CMP 100, GMP 100 and UMP 100 on the myotube atrophy in C2C12 myotube(n = 3. B . Representative images of myotubes. Scale bar = 100 μm. Green indicated Desmin staining, blue indicated DAPI staining of nuclei. C. Average diameters of myotubes(n = 3). D. mRNA expression of genes involved in muscular atrophy those are consistent with animal experiment (n = 3). Expression is shown relative to that of Model group. E. Protein expression of genes where are in Foxo, Akt/mTOR, and p53 pathways involved in sarcopenia which are consistent with animal experiment (n = 3). C‐D. Error bars indicated SEM. one‐way ANOVAs with LSD or Dunnetts’T3. # p < 0.05, ## p < 0.01, ### p < 0.001. # p < 0.05 versus Model group. Figure S6. The effect of NTs supplementation on the cell viability in C2C12 muscle atrophy cells. Error bars indicated SEM. one‐way ANOVAs with LSD or Dunnetts’T3. #p < 0.05, ##p < 0.01, ###p < 0.001. #p < 0.05 versus Model group. Figure S7. a‐f Quantitative analysis chart of common differential metabolites in different NTs‐treated groups vs Normal control group and Young control group [file JCSM-16-e70002-s002.docx]

**Supplemental information**

**Exogenous nucleotides supplementation** **attenuates age-related sarcopenia**

Xin Wu^1,2^, Rui Liu^1^, Na Zhu^3^, Xiujuan Wang^1^, Chan Wei^1^, Xiaoyang An, Meihong Xu^1^*, Yong Li^1^*

**Supplemental Materials and methods**

Cell culture and treatments

C2C12 cells were obtained from Zhejiang Meisen Cell Technology Co., LTD. They were cultured in DMEM with 1% penicillin/streptomycin and 10% fetal bovine serum at 37°C in a 5% CO2 incubator. When the density of cells reached 80%-90%, the cells were switched to DMEM-high glucose with 2% horse serum for differentiation. The cells became myotube cells after 4 days of differentiation. The myotubes were divided into the following groups: Control, Model group, AMP, CMP, GMP, UMP 50/100/200 μmol/L groups, and their mixture 50/100 μmol/L groups. The nucleotide mixture is composed of various nucleotide monomers in the following proportions, AMP: CMP: GMP: UMP = 22.80: 25.80: 30.20: 20.40. ^17^ H_2_O_2_ and intervention doses were based on literature, and cell cytotoxicity ruled out by CCK8 assay. After 48 hours, myotubes were either fixed for immunofluorescence or collected for biochemical studies.

*RNA-seq and analysis*

Following the manufacturer's instructions，total RNA was extracted from quadriceps (QUAD) muscles with TRIzol reagent (TIANGEN, China) and the integrity and total amount of RNA were accurately detected using Agilent 2100 bioanalyzer.The specific experimental operation and analytical methods are provided in the Supplementary Materials. The starting material for library construction was total RNA, from which poly(A)-tailed mRNA was enriched using Oligo(dT)-coated magnetic beads. The isolated mRNA was then randomly fragmented in the presence of divalent cations within a Fragmentation Buffer. Using the fragmented mRNA as a template and random hexamers as primers, the first strand of cDNA was synthesized in an M-MuLV reverse transcriptase reaction system. Following this, the RNA template was degraded using RNase H, and the second strand of cDNA was synthesized using DNA Polymerase I in the presence of dNTPs. After purification, the double-stranded cDNA underwent end repair, A-tailing, and adapter ligation. AMPure XP beads were used to select cDNA fragments ranging from approximately 370 to 420 bp. These selected fragments were subjected to PCR amplification and subsequently purified again using AMPure XP beads to obtain the final library. After the library is constructed, Qubit2.0 Fluorometer is used for preliminary quantification and the library is diluted to 1.5ng/ul. Then, Agilent2100bioanalyzer is used to detect the insert size of the library. qRT-PCR can accurately quantify the effective concentration of the library (the effective concentration of the library is higher than 2nM) to ensure the quality of the library. After the library check qualified, the libraries were sequenced on the Illumina novaseq 6000 platform. The basic principle of sequencing is sequencing by synthesis. Four kinds of fluorescently labeled dNTP, DNA polymerase and joint primers were added to the sequenced flowcell for amplification. When each sequencing cluster extended the complementary chain, each fluorescently labeled dNTP was added to release corresponding fluorescence. The sequencer captured the fluorescence signal and converted the optical signal into sequencing peak through computer software. Thus, the sequence information of the fragment to be tested can be obtained. In the data analysis stage, data quality control is carried out. In order to ensure the reliability of data analysis, the original data needs to be filtered. These include reads with adapter, reads containing N(N indicates that base information cannot be determined) and low-quality reads(Qphred ≤reads where base number of 20 accounts for more than 50% of the total read length). At the same time, the contents of Q20, Q30 and GC of cleandata were calculated. All subsequent analyses were based on high quality analysis conducted by cleandata. Sequence alignment to the reference genome: HISAT2v2.0.5 was used to construct the index of the reference genome, and HISAT2v2.0.5 was used to compare the paired terminal cleanreads with the reference genome. Quantification of gene expression levels: The software featureCounts (version 1.5.0-p3) was employed to calculate the number of reads mapped to each gene. Subsequently, the FPKM (Fragments Per Kilobase of transcript per Million mapped reads) values for each gene were computed based on the gene length and the number of reads mapped to that gene. FPKM represents the expected number of fragments per kilobase of transcript sequence per million base pairs sequenced, taking into account both sequencing depth and gene length, which is currently one of the most commonly used methods for estimating gene expression levels.

Differential expression analysis: For samples with biological replicates, differential expression analysis between two comparison groups was conducted using the DESeq2 package (version 1.30.1) in R. DESeq2 provides a statistical framework for determining differential expression in digital gene expression data using a model based on the negative binomial distribution. The Benjamini and Hochberg method was applied to adjust the resulting p-values to control the false discovery rate. Genes with a p-value < 0.05 identified by DESeq2 were designated as differentially expressed genes (DEGs). Additionally, a volcano plot was generated to illustrate the distribution of DEGs, and heatmaps of expression levels for the top 10 genes in each group of DEGs were created. The volcano plot was constructed using the ggplot2 package in R, while the gene expression heatmaps were produced using the pheatmap package in R.

*Metabolomic Profiles and Data Processing*Metabolomic profiles (n = 6/group) were identified using a High Throughput Targeted Quantification Kit (HM350, BGI, Shenzhen, China). Extensor digitorum longus（EDL） muscle tissue was mixed with a 50% water/methanol solution containing an internal standard for quality control. After centrifugation, the supernatant was analyzed using LC-MS/MS on a QTRAP 6500+ (SCIEX, USA). Chromatographic parameters included a BEH C18 column and Electron Spray Ionization (ESI) source. The concentration of metabolites was calculated using the integral peak area and standard curve. Differential metabolites were defined according to the following criteria: ratio >= 1.2 or ratio <= 0.83, adjusted p value < 0.05. Bioinformatics analyses were then performed, including Kyoto Encylopaedia of Genes and Genomes (KEGG) pathway classification. Sample preparation: Take the Extensor digitorum longus（EDL） muscle tissue samples and QC, add 140ul of 50% water/methanol solution, centrifuge to get the supernatant after crushing; Preparation of the standard song: take HM350 mixed standard, serially dilute, and prepare the standard song; Carry out derivatization reaction of sample, QC and Biaoqu song; Use HM350 diluent for dilution; Centrifuge at 12000r/min, 4℃for 10min, the supernatant was taken for LC-MS/MS analysis. LC-MS/MS detection and analysis. The analytical instrument for this experiment was LC-MS QTRAP 6500+ (SCIEX). Liquid chromatographic parameters. Chromatographic column: BEH C18 (2.1mm x 10cm, 1.7um, waters); Mass spectrometry parameters. Ion source: ESI+/ESI. Data statistics software and methods4.1 Method for calculating the concentration of metabolites. In MultiQuant software (SCIEX, USA), the default parameters are used for automatic identification and integration of each MRM transition (ion pair), and manual inspection is assisted. Data statistical method: Spearman correlation coefficient is applicable to continuous variables as well as discrete ordered variables. Spearman correlation coefficient between the two variables is equivalent to the Pearson correlation coefficient between the ranks of the two variables, so that the nonlinear correlation between variables is transformed into the linear correlation between their corresponding ranks. Therefore, Spearman correlation coefficient can detect more complex correlation, providing clues and evidence for further research and linearization. In this project, R package corr.test was used to achieve Spearman correlation analysis. Difference analysis method Univariate analysis is the simplest and most commonly used method for analyzing experimental data. In the analysis of differential metabolites between two groups of samples, the commonly used univariate analysis methods include Ratio analysis and t-test. We combined these two methods for differential metabolite analysis. In this project R package metaX was used for difference analysis.

**Supplemental Figures**

**Figure s1**

**
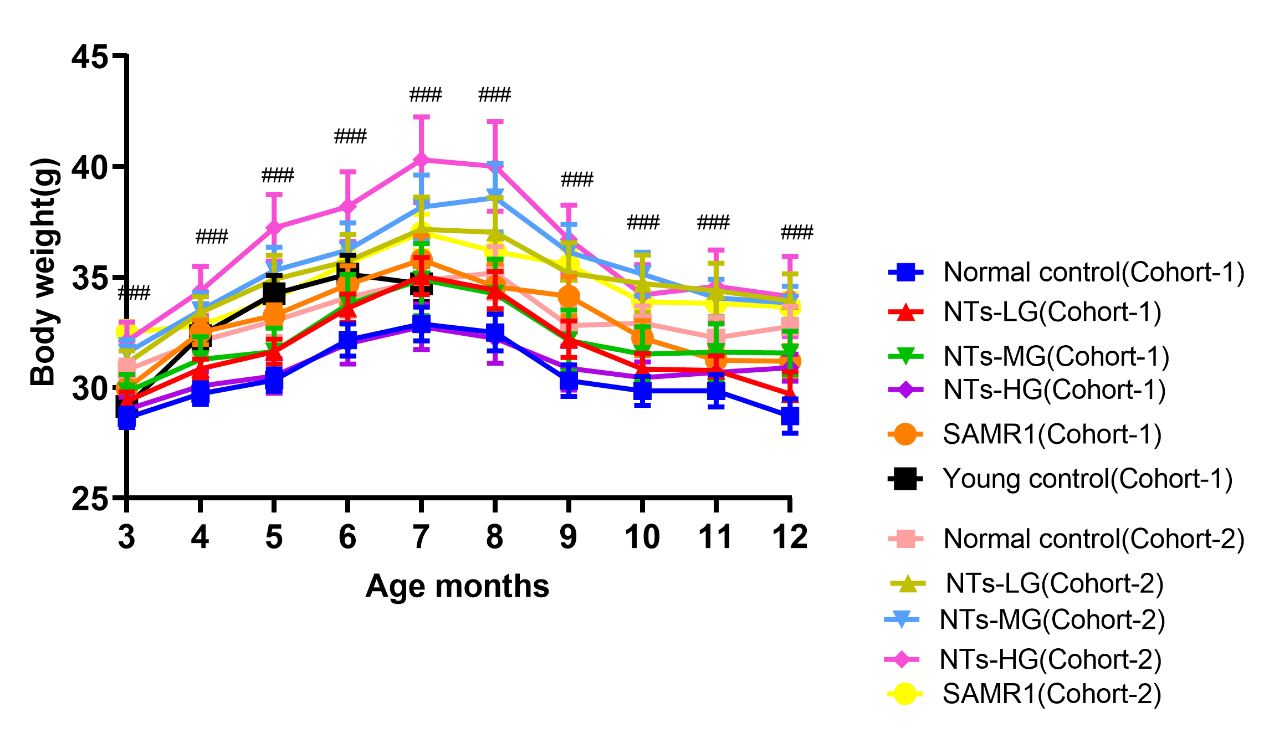
**

Effects of NTs supplementation on body weight in two cohorts. ^###^p < 0.001. ^###^p < 0.001 Cohort-2 versus Cohort-1.

**Figure s2**

**
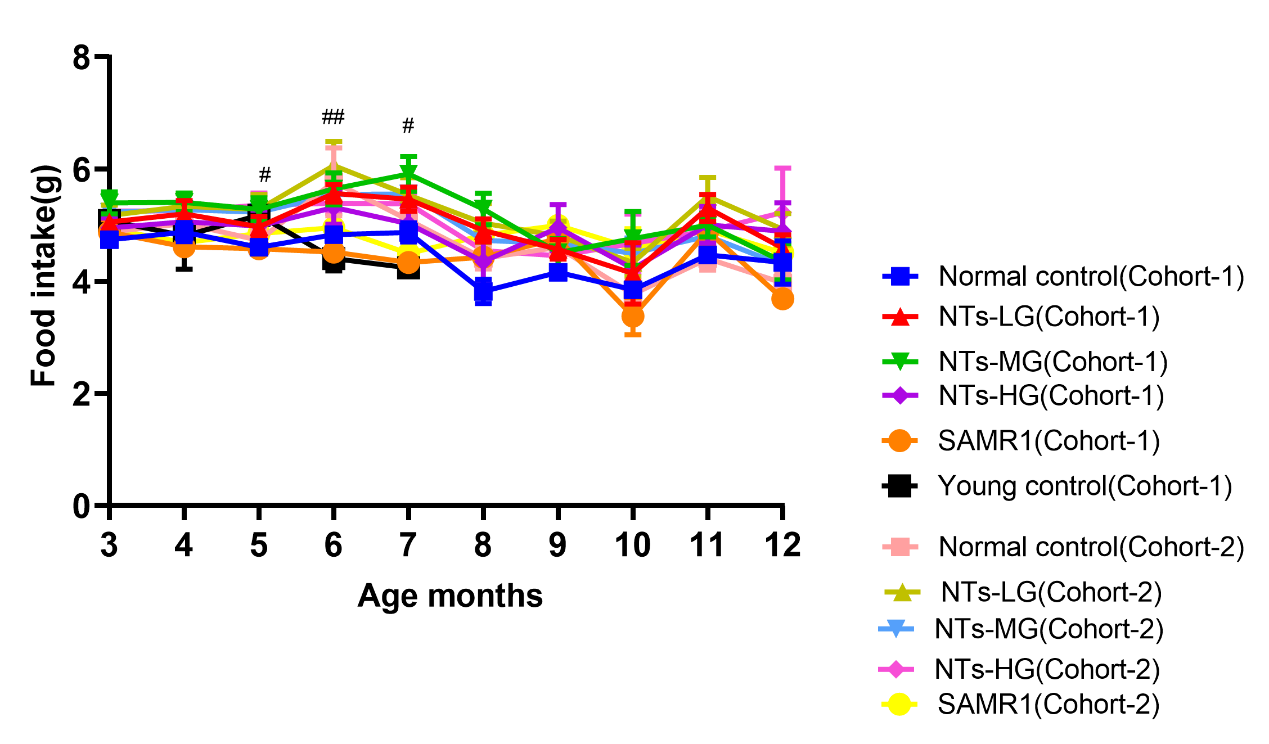
**

Effects of NTs supplementation on food intake(g/day) in two cohorts. ^#^p < 0.05, ^##^p < 0.01

**.** ^#^p < 0.05, ^##^p < 0.01 Cohort-2 versus Cohort-1**.**

**Figure S3**


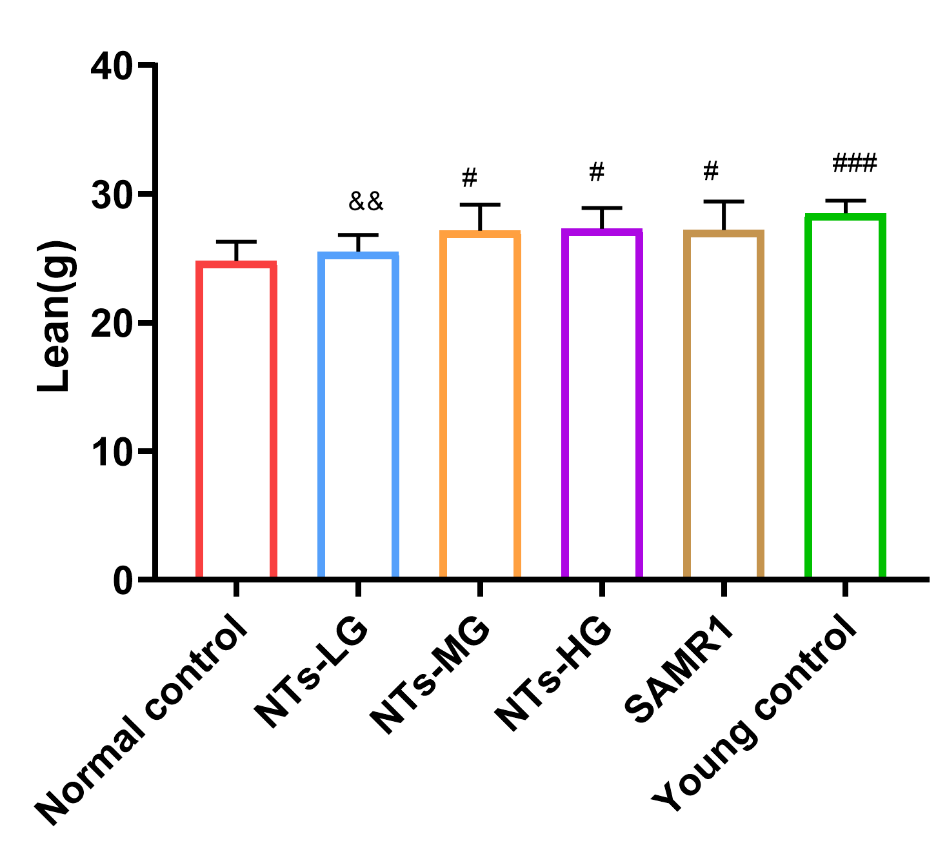


The effect of NTs supplementation on lean mass in SAMP8 mice. Error bars indicated SEM. one-way ANOVAs with LSD or Dunnetts’T3(D-G). ^#^p < 0.05, ^###^p < 0.001. ^#^p < 0.05 versus Normal control group. ^&&^p < 0.01 versus Young control group

**Figure S4**


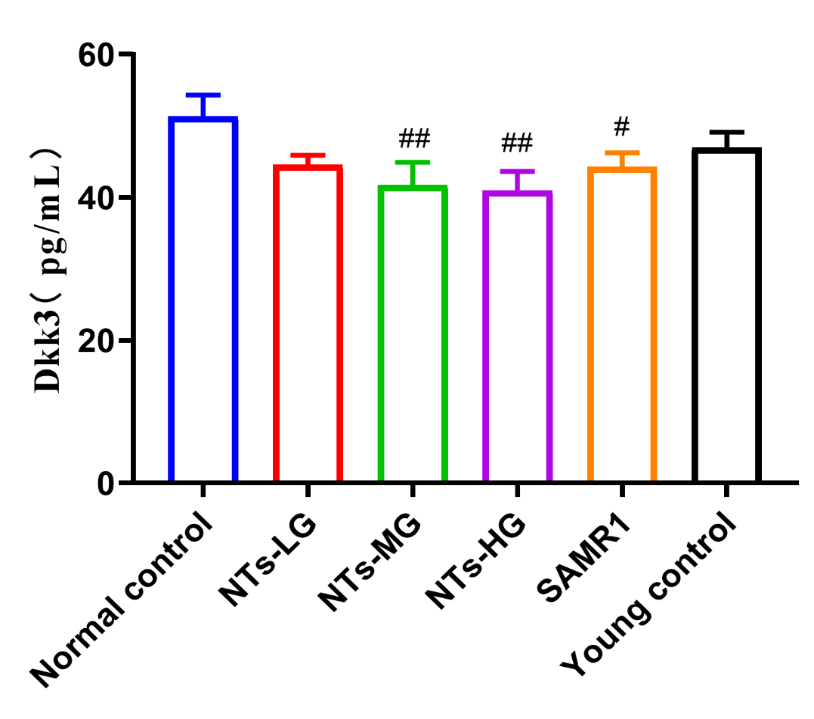


The effect of NTs supplementation on serum Dkk3 concentration in SAMP8 mice. Error bars indicated SEM. one-way ANOVAs with LSD or Dunnetts’T3(D-G). ^#^p < 0.05, ^##^p < 0.01,  ^###^p < 0.001. ^#^p < 0.05 versus Normal control group.

**S5**

**
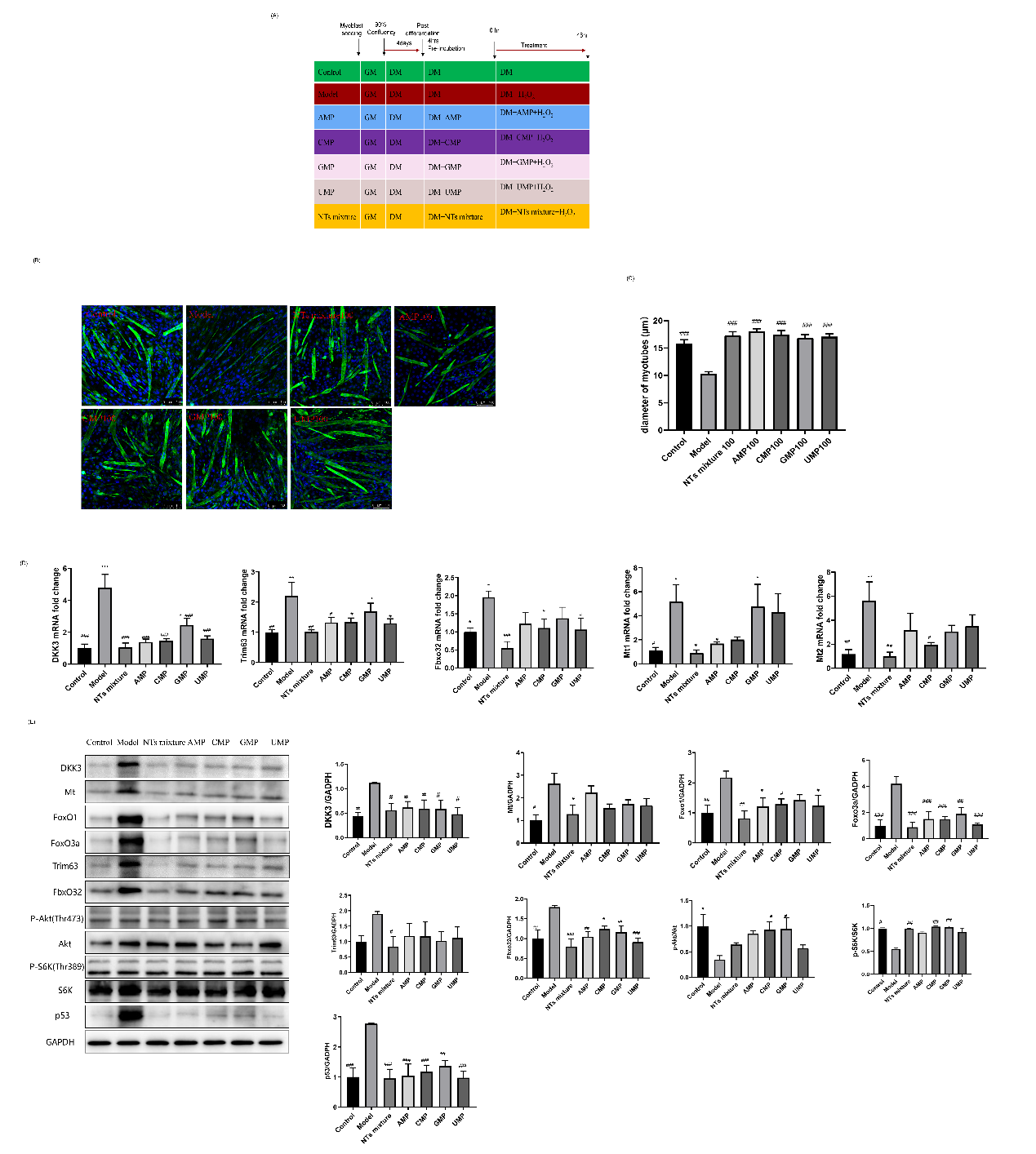
**

NTs ameliorates muscular atrophy in cultured C2C12 cells

A. Experimental timeline of C2C12 cells. B. Impact of NTs mixture 100, AMP 100, CMP 100, GMP 100 and UMP 100 on the myotube atrophy in C2C12 myotube（n=3. B . Representative images of myotubes. Scale bar = 100 μm. Green indicated Desmin staining，blue indicated DAPI staining of nuclei. C. Average diameters of myotubes（n=3）. D. mRNA expression of genes involved in muscular atrophy those are consistent with animal experiment (n= 3). Expression is shown relative to that of Model group. E. Protein expression of genes where are in Foxo, Akt/mTOR, and p53 pathways involved in sarcopenia which are consistent with animal experiment (n= 3). C-D. Error bars indicated SEM. one-way ANOVAs with LSD or Dunnetts’T3. ^#^p < 0.05, ^##^p < 0.01, ^###^p < 0.001. ^#^p < 0.05 versus Model group

**Figure S6**


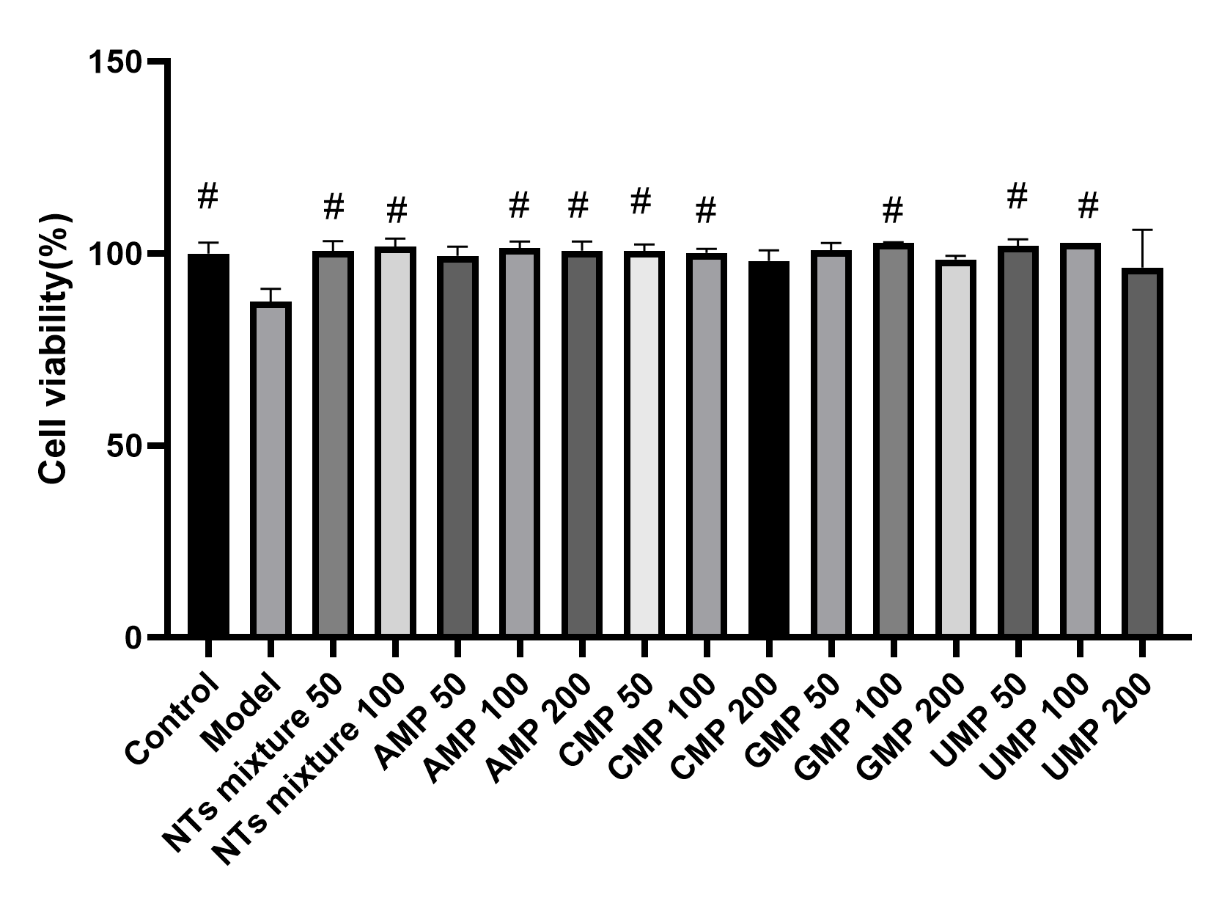


The effect of NTs supplementation on the cell viability in C2C12 muscle atrophy cells. Error bars indicated SEM. one-way ANOVAs with LSD or Dunnetts’T3(D-G). ^#^p < 0.05, ^##^p < 0.01, ^###^p < 0.001. ^#^p < 0.05 versus Model group

**Figure S7**


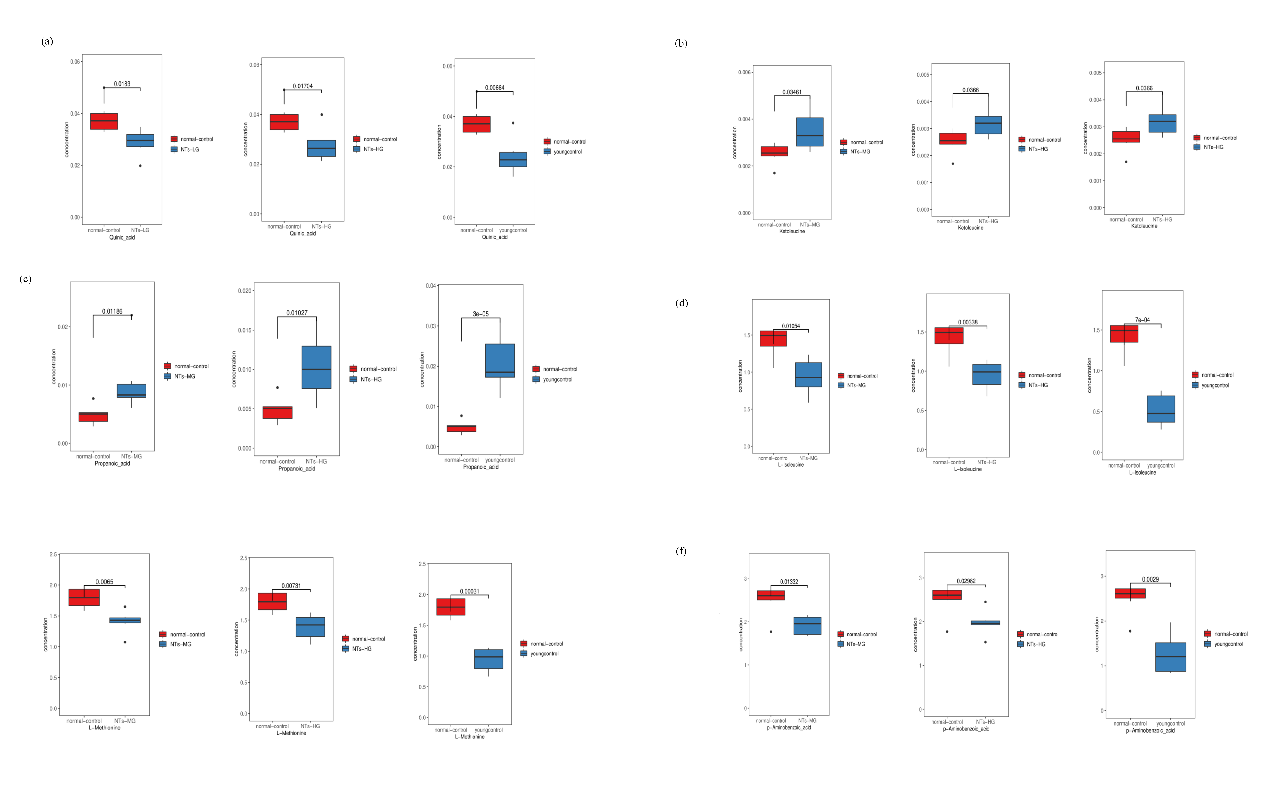
a-f Quantitative analysis chart of common differential metabolites in different NTs-treated groups vs Normal control group and Young control group vs Normal control group such as Ketoleucine propanoic_acid, L-Isoleucine, L-Methionine, p-Aminobenzoic_acid and Qunic_acid in different groups.


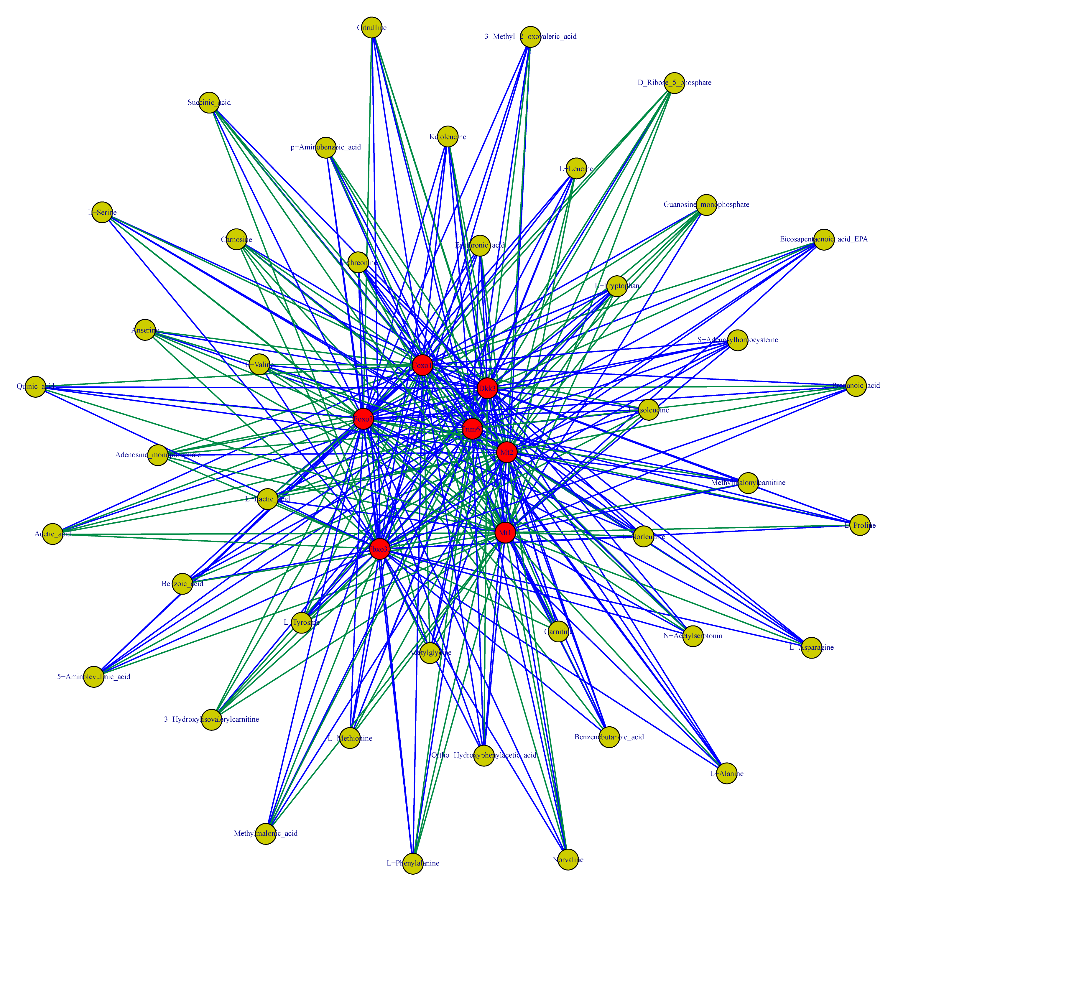
**Figure S8**

Network analysis of differential metabolites and target genes in the Young control group vs Normal control group

Table S1

Real-time qPCR primer sequences in this study

| Gene name | Forward sequences | Reverse sequences |
| --- | --- | --- |
| *Dkk3* | CAGCTCTCAACTACCCTCAGG | ACCTCAGAGGACGTTTTAGCA |
| *Fbxo32* | CAGCTTCGTGAGCGACCTC | GGCAGTCGAGAAGTCCAGTC |
| *Trim63* | GTGTGAGGTGCCTACTTGCTC | TGAGAGATGATCGTCTGCACT |
| *MT1* | AAGAGTGAGTTGGGACACCTT | CGAGACAATACAATGGCCTCC |
| *MT2* | GCCTGCAAATGCAAACAATGC | AGCTGCACTTGTCGGAAGC |
| *CLO1A1* | CCTCAGGGTATTGCTGGACAAC | CAGAAGGACCTTGTTTGCCAGG |
| *MyoD* | GCACTACAGTGGCGACTCAGAT | TAGTAGGCGGTGTCGTAGCCAT |
| *TNF-α* | GGTGCCTATGTCTCAGCCTCTT | GCCATAGAACTGATGAGAGGGAG |
| *GAPDH* | ACCCAGAAGACTGTGGATGG | ACACATTGGGGGTAGGAACA |

| Table S2 Antibody | | |
| --- | --- | --- |
| Antibody | source | identifier |
| P-Akt,dil:1/2000 | CST | Cat#:4060 |
| Akt,dil:1/2000 | Proteintech | Cat#:10176-2-AP |
| p-p70,dil:1/1000 | CST | Cat#:9234 |
| p70,dil:1/2000 | Proteintech | Cat#:14485-1-AP |
| Dkk3,dil:1/1000 | Abcam | Cat#:ab187532 |
| Fbxo32,dil:1/2000 | Abcam | Cat#:ab168372 |
| Trim63,dil:1/1000 | Proteintech | Cat#:55456-1-AP |
| mt1，mt2，dil:1/1000 | Abcam | Cat#:ab192385 |
| Foxo3a,dil:1/1000 | CST | Cat#:2497S |
| Foxo1,dil:1/1000 | CST | Cat#:2880S |
| p53,dil:1/1000 | CST | Cat#:2524S |
| HRP-GAPDH,dil:1/5000 | Proteintech | Cat#:HRP-60004 |
| Alexa Fluor 488,dil:1/800 | Invitrogen | Cat#:A11070 |
| Alexa Fluor 568，dil:1/800 | Invitrogen | Cat#:A11019 |
| Anti-Laminin Antibody,dil:1/500 | Sigma | Cat#:AB2034 |
| anti-MyHC I | DSHB | Cat#:BA-D5-S |
| anti-MyHC Iib | DSHB | Cat#:BF-F3 |
| Anti-Desmin antibody ,dil:1/1000 | Abcam | Cat#:ab15200 |

Table S3

The differential metabolites in the skeletal muscle of SAMP8 mice in the Young control group vs normal control group

| metabolite | ratio | VIP | P value |
| --- | --- | --- | --- |
| \| 5-Aminolevulinic_acid \| \| --- \| \| Acetylglycine \| \| Benzenebutanoic_acid \| \| Benzoic_acid \| \| Citrulline \| \| Eicosapentaenoic_acid_EPA \| \| L-Alanine \| \| L-Asparagine \| \| L-Isoleucine \| \| L-Leucine \| \| L-Methionine \| \| L-Norleucine \| \| L-Phenylalanine \| \| L-Proline \| \| L-Serine \| \| L-threonine \| \| L-Tryptophan \| \| L-Tyrosine \| \| L-Valine \| \| Norvaline \| \| Ortho-Hydroxyphenylacetic_acid \| \| p-Aminobenzoic_acid \| \| Quinic_acid   \| 3-Hydroxylisovalerylcarnitine \| \| --- \| \| 3-Methyl-2-oxovaleric_acid \| \| Acetic_acid \| \| Adenosine_monophosphate \| \| Anserine \| \| Carnitine \| \| Carnosine \| \| D_Ribose_5_phosphate \| \| Erythronic_acid \| \| Guanosine_monophosphate \| \| Ketoleucine \| \| L-Lactic_acid \| \| Methylmalonic_acid \| \| Methylmalonylcarnitine \| \| N-Acetylserotonin \| \| Propanoic_acid \| \| S-Adenosylhomocysteine \| \| Succinic_acid \| \| | \| \| 0.731 \| \| --- \| \| 0.721 \| \| 0.756 \| \| 0.382 \| \| 0.559 \| \| 0.636 \| \| 0.708 \| \| 0.636 \| \| 0.364 \| \| 0.708 \| \| 0.525 \| \| 0.685 \| \| 0.764 \| \| 0.739 \| \| 0.687 \| \| 0.737 \| \| 0.650 \| \| 0.628 \| \| 0.651 \| \| 0.568 \| \| 0.743 \| \| 0.505 \| \| 0.629 \| \| \| \| --- \| --- \| --- \| --- \| --- \| --- \| --- \| --- \| --- \| --- \| --- \| --- \| --- \| --- \| --- \| --- \| --- \| --- \| --- \| --- \| --- \| --- \| --- \| --- \| --- \| \| 1.465 \| \| 1.385 \| \| 1.293 \| \| 1.513 \| \| 1.276 \| \| 1.366 \| \| 1.328 \| \| 1.683 \| \| 1.402 \| \| 4.166 \| \| 1.497 \| \| 1.213 \| \| 12.024 \| \| 1.566 \| \| 1.297 \| \| 4.280 \| \| 1.350 \| \| 26.028 \| | \| 1.043 \| \| --- \| \| 1.108 \| \| 0.901 \| \| 2.591 \| \| 1.536 \| \| 1.446 \| \| 1.410 \| \| 1.614 \| \| 2.736 \| \| 1.339 \| \| 2.182 \| \| 1.570 \| \| 1.150 \| \| 1.102 \| \| 1.351 \| \| 1.095 \| \| 1.570 \| \| 1.792 \| \| 1.561 \| \| 1.909 \| \| 1.093 \| \| 2.090 \| \| 1.434 \| \| 1.235 \| \| 0.811 \| \| 0.993 \| \| 1.529 \| \| 0.974 \| \| 1.035 \| \| 0.951 \| \| 1.514 \| \| 1.027 \| \| 2.672 \| \| 0.925 \| \| 0.922 \| \| 2.453 \| \| 1.205 \| \| 0.818 \| \| 3.097 \| \| 0.773 \| \| 3.261 \| | \| 0.036 \| \| --- \| \| 0.023 \| \| 0.025 \| \| 0.041 \| \| 0.003 \| \| 0.012 \| \| 0.001 \| \| 0.001 \| \| 0.001 \| \| 0.001 \| \| 0.000 \| \| 0.000 \| \| 0.006 \| \| 0.009 \| \| 0.007 \| \| 0.013 \| \| 0.003 \| \| 0.001 \| \| 0.005 \| \| 0.000 \| \| 0.027 \| \| 0.003 \| \| 0.007 \| \| 0.007 \| \| 0.044 \| \| 0.034 \| \| 0.002 \| \| 0.024 \| \| 0.007 \| \| 0.025 \| \| 0.008 \| \| 0.016 \| \| 0.014 \| \| 0.045 \| \| 0.010 \| \| 0.039 \| \| 0.011 \| \| 0.039 \| \| 0.000 \| \| 0.019 \|   0.007 |

Table S4

The differential metabolites in the skeletal muscle of SAMP8 mice in the NTs supplementation group vs normal control group

| Group | metabolite | ratio | VIP | P value |
| --- | --- | --- | --- | --- |
| NTs-LG group  vs  normal control group | 2-Methylhexanoic_acid | 0.824314 | 1.207162 | 0.033837 |
|  | 2-Methylvaleric_acid | 0.625555 | 2.717215 | 0.027993 |
|  | 2-Phenylpropionate | 1.507342 | 1.777167 | 0.028148 |
|  | 3-Hydroxylisovalerylcarnitine | 1.450409 | 1.861511 | 0.025088 |
|  | Beta-Alanine | 0.732018 | 1.691201 | 0.04478 |
|  | Ethylmethylacetic_acid | 0.709486 | 1.47054 | 0.034974 |
|  | Isocaproic_acid | 0.828282 | 1.572038 | 0.021127 |
|  | Ortho-Hydroxyphenylacetic_acid | 0.769858 | 1.6024 | 0.015781 |
|  | Quinic_acid | 0.748646 | 1.52283 | 0.018302 |
|  | Sarcosine | 1.334561 | 1.559076 | 0.049671 |
| NTs-MG group  vs  normal control group | 2-Hydroxy-2-methylbutyric_acid | 0.517367 | 2.901555 | 0.033293 |
|  | 4-Hydroxyhippuric_acid | 0.499631 | 2.596123 | 0.022569 |
|  | Benzoic_acid | 0.416245 | 3.068504 | 0.002853 |
|  | Carnitine | 1.368963 | 1.899973 | 7.55E-04 |
|  | Isocaproic_acid | 0.798052 | 1.887893 | 9.93E-04 |
|  | Ketoleucine | 1.40823 | 1.408418 | 0.034614 |
|  | L-Asparagine | 0.792738 | 1.457445 | 0.009793 |
|  | L-Isoleucine | 0.66291 | 1.972823 | 0.010544 |
|  | L-Methionine | 0.78365 | 1.468333 | 0.006505 |
|  | L-Norleucine | 0.813014 | 1.538945 | 0.005727 |
|  | L-Pipecolic_acid | 1.273474 | 1.498553 | 0.032288 |
|  | p-Aminobenzoic_acid | 0.764804 | 1.33681 | 0.013322 |
|  | Propanoic_acid | 2.147619 | 2.727954 | 0.011858 |
| NTs-HG group  vs  normal control group | 3-Methyl-2-oxovaleric_acid | 1.357271 | 1.082273 | 0.012106 |
|  | 9-Pentadecenoic_acid | 0.531349 | 1.994071 | 0.027068 |
|  | Acetylglycine | 0.829732 | 0.745184 | 0.042783 |
|  | Ethylmethylacetic_acid | 0.755969 | 0.840979 | 0.044574 |
|  | Glyceraldehyde | 1.787162 | 1.947756 | 0.046259 |
|  | Ketoleucine | 1.301411 | 0.894277 | 0.036604 |
|  | L-Aspartic_acid | 0.716555 | 1.201819 | 0.028717 |
|  | L-Isoleucine | 0.67128 | 1.395327 | 0.003381 |
|  | L-Methionine | 0.774523 | 1.122125 | 0.007313 |
|  | L-threonine | 0.819548 | 0.921691 | 0.04007 |
|  | L-Tryptophan | 0.799839 | 1.099044 | 0.036873 |
|  | L-Valine | 0.784507 | 1.101625 | 0.009816 |
|  | N-Acetyl-L-aspartic_acid | 2.446545 | 2.438211 | 0.039906 |
|  | N-Acetylserine | 0.662629 | 1.584006 | 0.008272 |
|  | Norvaline | 0.776446 | 1.129178 | 0.038697 |
|  | p-Aminobenzoic_acid | 0.787967 | 0.877637 | 0.029817 |
|  | Propanoic_acid | 2.129737 | 2.159869 | 0.010267 |
|  | Quinic_acid | 0.722057 | 1.28328 | 0.017045 |
|  | Shikimic_acid | 2.306975 | 2.32293 | 0.019596 |
|  | Valeric_acid | 0.452767 | 2.86863 | 0.01783 |
