# Supplementary material for: Exogenous Nucleotides Supplementation Attenuates Age‐Related Sarcopenia
Source: J Cachexia Sarcopenia Muscle. 2025 Jul 31;16(4):e70002. doi: 10.1002/jcsm.70002 (PMC12313545; doi:10.1002/jcsm.70002)
Supplement: Supplementary file 2 — Data S1. Supplementary Information. [file JCSM-16-e70002-s001.docx]

**Exogenous nucleotides supplementation** **attenuates age-related sarcopenia**

Xin Wu^1,2^, Rui Liu^1^, Na Zhu^3^, Xiujuan Wang^1^, Chan Wei^1^, Xiaoyang An, Meihong Xu^1^*, Yong Li^1^*

Supplementary references

S1 Robinson S M, Jameson K A, Batelaan S F, Martin H J, Syddall H E, Dennison E M, et al. Diet and its relationship with grip strength in community-dwelling older men and women: the hertfordshire cohort study. J Am Geriatr Soc. 2008;56:84-90. doi:10.1111/j.1532-5415.2007.01478.x

S2 Wolfe R R. The underappreciated role of muscle in health and disease. Am J Clin Nutr. 2006;84:475-482. doi:10.1093/ajcn/84.3.475

S3 Gomes M D, Lecker S H, Jagoe R T, Navon A, Goldberg A L. Atrogin-1, a muscle-specific f-box protein highly expressed during muscle atrophy. Proc Natl Acad Sci U S A. 2001;98:14440-14445. doi:10.1073/pnas.251541198

S4 Bodine S C, Baehr L M. Skeletal muscle atrophy and the e3 ubiquitin ligases murf1 and mafbx/atrogin-1. Am J Physiol Endocrinol Metab. 2014;307:E469-E484. doi:10.1152/ajpendo.00204.2014

S5 Stitt T N, Drujan D, Clarke B A, Panaro F, Timofeyva Y, Kline W O, et al. The igf-1/pi3k/akt pathway prevents expression of muscle atrophy-induced ubiquitin ligases by inhibiting foxo transcription factors. Mol Cell. 2004;14:395-403. doi:10.1016/s1097-2765(04)00211-4

S6. Kamei Y, Miura S, Suzuki M, Kai Y, Mizukami J, Taniguchi T, et al. Skeletal muscle foxo1 (fkhr) transgenic mice have less skeletal muscle mass, down-regulated type i (slow twitch/red muscle) fiber genes, and impaired glycemic control. J Biol Chem. 2004;279:41114-41123. doi:10.1074/jbc.M400674200

S7 Derave W, Eijnde B O, Ramaekers M, Hespel P. Soleus muscles of samp8 mice provide an accelerated model of skeletal muscle senescence. Exp Gerontol. 2005;40:562-572. doi:10.1016/j.exger.2005.05.005

S8 Guo A Y, Leung K S, Siu P M, Qin J H, Chow S K, Qin L, et al. Muscle mass, structural and functional investigations of senescence-accelerated mouse p8 (samp8). Exp Anim. 2015;64:425-433. doi:10.1538/expanim.15-0025

S9 Hirofuji C, Ishihara A, Roy R R, Itoh K, Itoh M, Edgerton V R, et al. Sdh activity and cell size of tibialis anterior motoneurons and muscle fibers in samp6. Neuroreport. 2000;11:823-828. doi:10.1097/00001756-200003200-00033

S10 Ishida Y, Kiyokawa Y, Asai T, Oku N. Ameliorating effects of sphingomyelin-based liposomes on sarcopenia in senescence-accelerated mice. Biol Pharm Bull. 2016;39:786-793. doi:10.1248/bpb.b15-00915

S11 Guoquan Gao, Qiqun Tang. Biochemistry and Molecular Biology (10th Edition) [M]. Beijing: People's Medical Publishing House, 2024, 217.

S12 Van Buren C T, Rudolph F. Dietary nucleotides: a conditional requirement. Nutrition. 1997;13:470-472. doi:10.1016/s0899-9007(97)00103-2

S13 Kulkarni A D, Rudolph F B, Van Buren C T. The role of dietary sources of nucleotides in immune function: a review. J Nutr. 1994;124:1442S-1446S. doi:10.1093/jn/124.suppl_8.1442S

S14 Yamauchi K, Hales N W, Robinson S M, Niehoff M L, Ramesh V, Pellis N R, et al. Dietary nucleotides prevent decrease in cellular immunity in ground-based microgravity analog. J Appl Physiol (1985). 2002;93:161-166. doi:10.1152/japplphysiol.01084.2001

S15 Xu M, Liang R, Guo Q, Wang S, Zhao M, Zhang Z, et al. Dietary nucleotides extend the life span in sprague-dawley rats. J Nutr Health Aging. 2013;17:223-229. doi:10.1007/s12603-012-0399-z

S16 .Tressler R L, Ramstack M B, White N R, Molitor B E, Chen N R, Alarcon P, et al. Determination of total potentially available nucleosides in human milk from asian women. Nutrition. 2003;19:16-20. doi:10.1016/s0899-9007(02)00843-2

S17 Latres E, Amini A R, Amini A A, Griffiths J, Martin F J, Wei Y, et al. Insulin-like growth factor-1 (igf-1) inversely regulates atrophy-induced genes via the phosphatidylinositol 3-kinase/akt/mammalian target of rapamycin (pi3k/akt/mtor) pathway. J Biol Chem. 2005;280:2737-2744. doi:10.1074/jbc.M407517200
S18 Lecker S H, Jagoe R T, Gilbert A, Gomes M, Baracos V, Bailey J, et al. Multiple types of skeletal muscle atrophy involve a common program of changes in gene expression. Faseb J. 2004;18:39-51. doi:10.1096/fj.03-0610com
S19 Lecker S H, Jagoe R T, Gilbert A, Gomes M, Baracos V, Bailey J, et al. Multiple types of skeletal muscle atrophy involve a common program of changes in gene expression. Faseb J. 2004;18:39-51. doi:10.1096/fj.03-0610com

S20 Niehrs C. Function and biological roles of the dickkopf family of wnt modulators. Oncogene. 2006;25:7469-7481. doi:10.1038/sj.onc.1210054

S21 Stitt T N, Drujan D, Clarke B A, Panaro F, Timofeyva Y, Kline W O, et al. The igf-1/pi3k/akt pathway prevents expression of muscle atrophy-induced ubiquitin ligases by inhibiting foxo transcription factors. Mol Cell. 2004;14:395-403. doi:10.1016/s1097-2765(04)00211-4

S22 Escobar J, Frank J W, Suryawan A, Nguyen H V, Van Horn C G, Hutson S M, et al. Leucine and α-ketoisocaproic acid, but not norleucine, stimulate skeletal muscle protein synthesis in neonatal pigs. The Journal of Nutrition. 2010;140:1418-1424. doi:10.3945/jn.110.123042

S23 Cui, P.; Shao, W.; Huang, C.; Wu, C.J.; Jiang, B.; Lin, D. Metabolic derangements of skeletal muscle from a murine model of glioma cachexia. Skelet Muscle. 2019, 9, 3.
